# Metagenomic and cultivation-based description of a syntrophic butyrate-oxidizing bacterium from a thermophilic and high-ammonia biogas process

**DOI:** 10.1186/s12866-026-05457-y

**Published:** 2026-07-30

**Authors:** Malin Tiefensee, Nils Weng, Jonas A. Ohlsson, Maria Westerholm

**Affiliations:** Department of Molecular Sciences, Swedish University of Agricultural Sciences, Uppsala, Sweden

**Keywords:** Anaerobic digestion, Syntrophy, Syntrophic oxidation, Metagenome assembled genome, Functional gene analysis, *Syntrophothermus*

## Abstract

**Background:**

Ammonia inhibition in anaerobic digestion can lead to butyrate accumulation and reduced methane yield. Despite the importance of syntrophic butyrate oxidation in mitigating this effect, the microorganisms and interactions involved under high-ammonia conditions remain poorly understood. Here, we combine metagenomics and cultivation studies to describe a novel ammonia-tolerant syntrophic butyrate-oxidizing bacterium and its interactions with acetate-oxidizing bacteria and hydrogenotrophic methanogens enriched from a high-ammonia, thermophilic biogas process.

**Results:**

The enrichment culture degraded butyrate at rates of 0.12–0.47 mmol/day. Amplicon sequencing and phylogenetic analyses of a retrieved metagenome-assembled genome (MAG) assigned the putative syntrophic butyrate-oxidizing bacterium (SBOB) to the genus *Syntrophothermus*, for which we propose the provisional species name ‘*Candidatus* Syntrophothermus ammoniitolerans’. Metagenomic analyses revealed the genomic potential for β-oxidation and essential electron transfer pathways associated with syntrophic energy conservation. Furthermore, one additional MAG (MAG9) possessed a complete β-oxidation pathway but lacked key genes associated with reverse electron transfer, making its role as a SBOB uncertain. Acetate produced during butyrate oxidation was further oxidized by syntrophic acetate-oxidizing bacteria and ultimately converted to methane by hydrogenotrophic methanogens, illustrating a tightly coupled metabolic network that supports butyrate degradation under high-ammonia conditions. Three methanogenic MAGs, affiliated with the genera *Methanoculleus* and *Methanothermobacter*, were identified as potential hydrogen- or formate-consuming partners.

**Conclusions:**

Together, these results identify a novel syntrophic butyrate-oxidizing candidate that enables butyrate degradation under high-ammonia conditions via tightly coupled interactions with acetate-oxidizing bacteria and hydrogenotrophic methanogens, sustaining methane production under ammonia stress.

**Supplementary Information:**

The online version contains supplementary material available at 10.1186/s12866-026-05457-y.

## Introduction

Biogas is a renewable fuel produced through anaerobic digestion, a process that also generates digestate, a solid biofertilizer that recycles essential nutrients to arable land and serves as a low-emission alternative to synthetic fertilizers [1]. Many commonly used anaerobic digestion substrates are rich in proteins and amino acids, enhancing the agronomic value of the digestate [2] but also leading to elevated ammonia concentrations and an increased risk of process inhibition [3]. Process disturbance under ammonia-inhibitory conditions, typically occurring at 0.6–1.5 g NH_3_-N/L (or 2.5–11 g NH_4_^+^-N/L) at elevated temperatures (51–64 °C, pH 7.4–7.9) [4] initially manifests as acetate accumulation and reduced methane production, reflecting inhibition of acetate-utilizing (acetoclastic) methanogens [5, 6]. Following microbial acclimation, process recovery may occur through a shift to syntrophic acetate oxidation (SAO), in which ammonia-tolerant syntrophic acetate-oxidizing bacteria (SAOB) oxidize acetate to formate or hydrogen (H_2_) and carbon dioxide (CO_2_) with the resulting intermediates converted to methane (CH_4_) by hydrogenotrophic methanogens [7–10]. Shifts from acetoclastic methanogenic dominance to increased activity of the SAOB and hydrogenotrophic methanogens have been reported around 0.14–0.5 g/L NH_3_-N at mesophilic and 0.03–0.5 g/L NH_3_-N under thermophilic temperature conditions, respectively, and pH levels in the 7.5–8.0 range [4, 11]. Ammonia inhibition of butyrate-degrading microorganisms has been less extensively investigated. However, non-acclimated communities have been reported to be inhibited at concentrations above 0.3 g NH_3_-N/L under mesophilic conditions (37 °C, pH 7.5), resulting in prolonged lag phases, reduced degradation rates, and incomplete butyrate degradation with increasing ammonia concentrations [12].

The bacteria involved in butyrate degradation are referred to as syntrophic butyrate-oxidizing bacteria (SBOB). In low-ammonia anaerobic digestion systems, previous studies have identified SBOB within the phylum Bacillota, belonging to the genera *Syntrophomonas* (mesophilic) [13–19], *Syntrophothermus,* and *Thermosyntropha* (thermophilic) [20, 21]. In addition, a mesophilic SBOB has been identified in the phylum Thermodesulfobacteriota, belonging to the genus *Syntrophus* [22]. However, the identity of SBOB in high-ammonia systems remain largely unresolved. All characterized SBOB so far have been shown to utilize the β-oxidation pathway for butyrate oxidation [23–25]. In this pathway, oxidation of one molecule of butyrate theoretically yields two molecules of acetate, while the released electrons are transferred to syntrophic partners via hydrogen and/or formate. Under thermophilic conditions, the acetate formed via syntrophic butyrate oxidation (SBO) has been suggested to be degraded by SAOB [26].

The oxidation of butyrate and acetate (Eqs. 1–2) is thermodynamically unfavourable under standard conditions [27, 28], and for syntrophic acid oxidation to proceed [28], hydrogenotrophic methanogens must maintain low H_2_ partial pressures and formate concentrations (Eqs. 3–4) [8, 24]. The precise thresholds remain insufficiently studied, but formate levels up to 10 μM and H_2_ levels of 4–171 Pa have been reported in mesophilic syntrophic butyrate oxidation (SBO) cultures [29, 30]. Moreover, complete inhibition of butyrate degradation has been observed at H_2_ partial pressures above 2000 Pa in experiments involving formate perturbation [30].

1 $$\begin{aligned}&{\mathrm{C}}_{4}{\mathrm{H}}_{7}{\mathrm{O}}_{2}^{-}+ 2{\mathrm{H}}_{2}\mathrm{O}\to 2{\mathrm{C}}_{2}{\mathrm{H}}_{3}{\mathrm{O}}_{2}^{-}+{\mathrm{H}}^{+ }+2{\mathrm{H}}_{2}\\&\Delta\text G^{\circ\prime}=+48.3\ \mathrm{kJmol}^{-1}\end{aligned}$$

2 $$\begin{aligned}&{\mathrm{C}}_{2}{\mathrm{H}}_{3}{\mathrm{O}}_{2}^{-}+2{\mathrm{H}}_{2}\mathrm{O}\to 2{\mathrm{CO}}_{2}+ 3{\mathrm{H}}_{2}+{\mathrm{H}}^{+}\\&\Delta\text G^{\circ\prime}=+104.6\ \mathrm{kJmol}^{-1}\end{aligned}$$

3 $$\begin{aligned}&4{\mathrm{H}}_{2}+{\mathrm{C}\mathrm{O}}_{2}\to {\mathrm{C}\mathrm{H}}_{4}+2{\mathrm{H}}_{2}\mathrm{O}\\&\Delta\text G^{\circ\prime}=-135.0\ \mathrm{kJmol}^{-1}\end{aligned}$$

4 $$\begin{aligned}&2{\mathrm{H}}_{2}+0.5{\mathrm{CO}}_{2}\to 0.5{\mathrm{CH}}_{4}+ {\mathrm{H}}_{2}\mathrm{O}\\&\Delta\text G^{\circ\prime}=-67.5\ \mathrm{kJmol}^{-1}\end{aligned}$$

Consequently, SBOB must establish partnerships with hydrogenotrophic methanogens capable of maintaining formate and H₂ concentrations at sufficiently low levels. Partners of SBOB in co-cultures include the mesophilic genera *Methanospirillum*, *Methanococcus*, *Methanobacterium, Methanoculleus*, as well as the thermophilic genera *Methanocella* and *Methanothermobacter* [13, 16, 31–34]. In enrichment cultures, *Methanolinea* [35]*, Methanosarcina* [36], and *Methanoculleus* [37, 38] have also been suggested as syntrophic partners under mesophilic or thermophilic conditions. Under high-ammonia conditions, the methanogenic partner must not only maintain low formate and H_2_ levels but also tolerate ammonia stress. However, as with the limited understanding of SBOB taxonomy under high-ammonia conditions, the identity of their preferred hydrogen-consuming partners, as well as their interactions with acetate-utilizing microorganisms, remains unclear.

To address this knowledge gap, this study identifies and describes a novel ammonia-tolerant putative SBOB derived from enrichment cultures originating from high-ammonia biogas processes. Cultivation experiments were conducted to determine butyrate degradation rates and perform stoichiometric analyses under high-ammonia conditions. Furthermore, 16S rRNA gene amplicon sequencing and metagenome-assembled genome (MAG) analyses were employed to establish phylogenetic placement, reconstruct metabolic pathways, and evaluate potential syntrophic interactions.

## Material and methods

### Source of syntrophic communities

The enriched syntrophic communities in this study originate from a 1.1 L laboratory-scale bioreactor operated under thermophilic conditions (52 °C), as previously described [39]. The bioreactor was inoculated with digestate from a large-scale biogas plant treating food waste from households and industries and minor fractions of slaughterhouse waste. The source digester operated at 52 °C and contained 3.2 g/L NH_4_^+^-N, 0.6 g/L NH_3_, indicating that the microbial community was adapted to elevated ammonia levels. For two years, the bioreactor was continuously fed with anoxic, sterile, bicarbonate-buffered basal medium containing yeast extract (0.2 g/L), trace elements, vitamins and ammonium chloride (8.9 g/L, 0.17 M). L-cysteine HCl (0.5 g/L) and Na_2_S (0.24 g/L) were added as reducing agents and sodium propionate (9.6 g/L, 0.1 M) was added as the main carbon and energy source. During this period, butyrate remained below the detection limit. The reactor was subsequently switched to semi-batch operation and fed weekly with the same medium. Under these conditions, low concentrations of butyrate (2.3–4.6 mM) were occasionally detected, suggesting active butyrate formation and turnover. Following an additional two years of semi-batch operation, reactor biomass was transferred to batch assays supplied with sodium butyrate to investigate the butyrate-degrading community.

### Enrichment and cultivation of the syntrophic butyrate-oxidizing culture

Batch cultivations were established in 500 mL anaerobic flasks containing 250 mL anoxic basal medium and an N_2_:CO_2_ (80:20) headspace, as previously described [40]. Initially, the medium was supplemented with yeast extract and sodium butyrate (YB medium), and cultivation was conducted in triplicate. To further enrich the SBO community, subsequent transfers were performed in medium lacking yeast extract and supplemented with sodium butyrate (0B medium). In parallel, a single acetate-fed culture was established in medium without yeast extract and supplemented with sodium acetate as the sole carbon and energy source to identify microorganisms involved in acetate degradation. Reducing agents, vitamins, mineral solutions, and ammonium chloride were added as described above. All cultures were inoculated with biomass originating from the propionate-fed bioreactor described above [39] (Table 1) and incubated statically in the dark at 51 ± 1 °C. Within the duration of the present study, attempts to repeatedly transfer the 0B culture in medium without yeast extract were unsuccessful for reasons that remain unclear. Therefore, rather than continuing serial transfers, the enrichment was maintained as a single long-term culture to monitor community development and substrate conversion over time. Following depletion of the supplied substrate and its degradation products, the 0B culture was fed additional substrate (30 mM sodium butyrate) on days 330, 449, 488, 554, 629, 678, 764 and 848. To compensate for sampling losses and restore the culture volume, 75 mL of fresh 0B medium was added on day 488.

**Table 1:** Description of the enrichment cultures

Culture name	YB	0B	Acetate control
Replication type	Triplicate	Single	Single
Type	Experimental	Experimental	Control
Inoculum source	Propionate-fed bioreactor	Propionate-fed bioreactor	Propionate-fed bioreactor
Inoculum [v/v]	5%	5%	5%
Substrate	Sodium butyrate, 30 mM	Sodium butyrate, 30 mM	Sodium acetate, 30 mM
Yeast extract [g/L]	0.2	0	0
NH_4_Cl [M]	0.1	0.08	0.17
Duration [days]	95	972	NA
Number of degradation cycles	1	8	NA

*NA* Not available

### Analytical methods

Headspace CO_2_ and CH_4_ concentrations were quantified using gas chromatography equipped with flame ionization/thermal conductivity detectors, as previously described [40]. On day 805 of the 0B cultivation, but after the finalization of the YB cultures, the carrier gas of the instrument was changed from He to Ar. Headspace pressure was assessed with a pressure meter (GMH 3111, Gresinger). When the pressure exceeded 1000 mbar, the bottles were vented and an additional gas sample was taken to quantify the amount of CH_4_ and CO_2_ removed. Molar gas quantities were calculated using the ideal gas law (Eq. 5),

5 $$\mathrm{pV}=\mathrm{nRT}$$

where p is bottle pressure [Pa], V the headspace volume [m^3^], R = 8.314 Jmol^−1^ K^−1^, and T = 52 °C (325.15 K).

Lactate and volatile fatty acids (VFAs) (acetate, propionate, iso-butyrate, butyrate, iso-valerate, valerate) in the YB cultures were quantified by high-performance liquid chromatography (HPLC) using a refractive index detector having a detection limit of 0.1 g/L as previously described [40]. For 0B and the acetate control, sample preparation was largely identical, except that 200 μL sample volume was analysed and the centrifugation step was omitted. These samples were analysed using an upgraded HPLC system equipped with a UV detector [41], with the same detection limit. To ensure comparability between datasets generated with the two systems, calibration standards covering the relevant concentration range were analysed during method implementation, yielding comparable calibration curves and quantification results.

For the YB cultures, VFA degradation rates were determined by linear regression of concentration versus time during the near-linear phase of degradation for each replicate and are reported as the mean across replicates. For the 0B culture, butyrate degradation in each depletion cycle was described using a modified Gompertz model adapted for substrate depletion [42] (Eq. 6), with the first observation in each cycle defined as t = 0,

6 $$\mathrm{S}\left(\mathrm{t}\right)={\mathrm{S}}_{0}+ {\mathrm{S}}_{\mathrm{m}\mathrm{a}\mathrm{x} }\cdot \exp\left\{-\mathrm{e}\mathrm{x}\mathrm{p}\left[\frac{{\mathrm{R}}_{\mathrm{m}\mathrm{a}\mathrm{x},\mathrm{S}}\cdot \mathrm{e}}{{\mathrm{S}}_{\mathrm{m}\mathrm{a}\mathrm{x}}}\cdot \left(\uplambda -\mathrm{t}\right)+1\right]\right\}$$

where S(t) is the butyrate amount at time t [days], S_0_ is the initial butyrate amount [mmol], S_max_ is the total amount of butyrate degraded during the cycle, R_max,S_ is the maximum degradation rate [mmol/day] and λ represents the lag time. For each cycle, S_0_ and S_max_ were fixed from the observed start and end values, whereas the other parameters were estimated by nonlinear least-squares fitting. The maximum degradation rate (R_max,S_) is presented as the mean across depletion cycles.

Samples for pH were taken concurrently with VFA sampling from the 0B culture and analysed with a SevenCompact S220 pH meter equipped with an InLab micro-Pro-ISM pH sensor (Mettler Toledo, Ohio, USA). pH was not assessed during growth of the YB cultures. The ammonia concentration, [NH_3_], was calculated using the equation defined by Hansen et al. [43]

7 $$\left[{\mathrm{N}\mathrm{H}}_{3}\right]=\frac{\left[{\mathrm{N}\mathrm{H}}_{4}^{+}\right] }{1+ \frac{{10}^{-\mathrm{p}\mathrm{H}}}{{10}^{-(0.09018+\frac{2729.92}{\mathrm{T}})}}}$$

where NH_4_^+^ [g/L] was derived from the initial NH_4_Cl concentration, while pH and temperature (T) represent measured values during cultivation.

Cell morphology and autofluorescence of coenzyme F_420_ were examined and fluorescence–brightfield composite micrographs were taken using a Lumascope LS720 (Etaluma Inc, California, USA) as previously described [44].

### DNA extraction and quality control for sequencing using Illumina and Nanopore MinION

DNA for 16S rRNA gene analysis was extracted from 2 mL of liquid culture using the DNeasy Blood & Tissue kit (Qiagen, Hilden, Germany) according to the manufacturer’s instructions. Long-fragment DNA for Nanopore sequencing was extracted twice from 16 and 24 mL of liquid culture using NucleoBond AXG columns and NucleoBond Buffer set III as previously described [45]. Duplicate DNA extractions were performed, where one sample was treated according to the original protocol, whereas the second sample had increased incubation times (2 h at 37 °C with lysozyme and proteinase K, followed by 2 h of incubation at 50 °C). DNA quantification was performed using a Qubit 3.0 fluorometer (FischerScientific, UK). The long-fragment DNA was additionally analysed with an Agilent 4150 TapeStation (Santa Clara, USA) according to the Genomic DNA ScreenTape analysis protocol to ensure that the DNA integrity number and fragment length were sufficient for further analysis. DNA Integrity Number of the samples was assessed, and the samples were also analysed using a Nanodrop Spectrophotometer, where the A260/280 nm ratios were manually inspected to select the method yielding the highest amount of non-contaminated, high-quality DNA.

### 16S rRNA gene amplification sequencing

For 16S rRNA gene sequencing, samples from the YB cultures were taken approximately weekly between days 4–88. The sequence library was prepared using the primers 341 F and 805R which were complemented with sample-specific barcodes [46, 47]. The first PCR reaction included 3 μL of DNA extract (< 13.89 ng of DNA), and the thermocycler was programmed as follows: 30 s at 98 °C, followed by 15 cycles of 10 s at 98 °C, 30 s at 66 °C and 30 s at 72 °C, and a final extension of 2 min at 72 °C. PCR products were stored at 4 °C until further analysis. PCR product cleaning was conducted using AMPure HighPrep beads (Beckman Coulter Life Sciences, California, USA) with some modifications to the protocol provided by the manufacturer: the volume of the magnetic beads was 0.8 times the sample volume, the separation time on the magnetic plate was increased to 5 min, and the elution volume was 20 μL. For the second PCR, 1.25 μL [10 pM] of each of the sample-specific barcoded forward and reverse primers were used, and the number of cycles was shortened to 12. Following quantification, 10 ng DNA per sample was pooled for sequencing. Next-generation amplicon sequencing was performed using Illumina MiSeq technology [48] with Reagent Kit v3. Five DNA samples from the 0B culture and the acetate control culture were submitted to Novogene Co. (2025) for amplicon sequencing of bacterial and archaeal 16S rRNA genes. The DNA extracts were either reused from the long-read extraction (day 474, 510 and 552 (SBOB)) or separately extracted using the Blood&Tissue kit as presented above (day 196 (SAOB) and 971 (SBOB)). Bacterial 16S rRNA genes were amplified using the primers 515 F and 806R, targeting the V4 region. Archaeal 16S rRNA genes were amplified using the primers Arch519F and Arch915R, targeting the V4–V5 region [49–52].

### Quantitative PCR

Quantitative PCR (qPCR) was performed to determine the 16S rRNA gene copy number of methanogens at all DNA extraction time points for cultures YB, 0B, and the acetate control. The primer pairs MMB282F/MMB832R and MBT857F/MBT1196R were used to determine the 16S rRNA gene abundance of methanogens of the order Methanomicrobiales and Methanobacteriales, respectively [53]. The qPCR protocol was as follows: 7 min at 95 °C, followed by 40 cycles of 95 °C for 40 s, annealing at 66 °C (Methanomicrobiales) or 61 °C (Methanobacteriales) for 1 min, and 72 °C for 40 s, and melting curve analysis at 95 °C for 15 s, followed by 1 min at 55 °C and finally at 95 °C for 1 s. The reactions were carried out using QuantStudio™ 5 (ThermoFisher). Results were normalized to sample DNA content and expressed as absolute gene copies per ng DNA.

### Metagenome sequencing

Samples for metagenome sequencing were taken from culture 0B on days 474, 510 and 552. Sequence library preparation was performed thrice with the SQK-NBD114.24 kit from Oxford Nanopore, each time using 1000 ng of long-fragment DNA as starting material. While two runs followed the standard manufacturer's protocol, the third was adapted by prolonging all incubation steps by 30–50% and substituting the kit beads with AMPure HighPrep beads (Beckman Coulter Life Sciences, California, USA). In all cases, multiple reactions of the same sample were performed and barcoded with the same barcode before being pooled according to protocol. When two different DNA extraction methods were compared, two reactions for each method were prepared and pooled. MinION sequencing was performed with a version 10.4.1 flow cell, run for 43–71 h with default parameters, live high-accuracy barcoding with 450 base pair per second and a Q-score of 9 for Guppy (v 7.2.13) [54]. Illumina metagenome sequencing was performed by ScilifeLab (Uppsala, Sweden) using 200 ng of the long-fragment DNA extract from day 552. The sequencing library was prepared with the SNP&SEQ platform using the ThruPLEX DNA-seq library preparation kit (Takara Bio). The SNP&SEQ platform performed cluster generation and 150 cycles of paired-end sequencing in one lane of a 10B flow cell using the NovaSeqX Plus system and XLEAP-SBS sequencing chemistry (Illumina Inc. 2024).

### Data analyses

For the YB cultures, 16S rRNA gene amplicons were trimmed to 280 bp by removing adapters and primers using Cutadapt (v 4.1) [55], whereas for the 0B culture, adapter and primer trimming had already been performed by the sequencing provider. All reads were processed using the DADA2 pipeline (v 1.36.0) [36, 56–58] in R (v 4.5.0) with otherwise default parameters. Based on quality profile inspection, optimal truncation lengths were set to 265 bp (forward) and 220 bp (reverse) for the YB cultures, and 220 bp for both forward and reverse reads for the 0B culture. Taxonomic assignment of the resulting amplicon sequence variants was performed using the IDTAXA [59] classifier against the SILVA database (v138.2) [60]. For downstream visualization, amplicon sequence variants annotated as *Incertae sedis* were treated as unassigned at the respective taxonomic rank. Individual sequences were analysed using the Basic Local Alignment Search Tool (BLAST) [61].

Nanopore sequencing, data binning and annotation were conducted by Nanozoo (Germany). Raw Nanopore sequencing data were basecalled and demultiplexed using Guppy (v 6.0.1) [54] and filtered using Filtlong (v 0.2.0) [62]. Reads from the different Nanopore runs were merged with the Illumina metagenome data and all the presented MAGs were obtained from the joint data. NanoPlot (v1.42.0) was used for quality assessment [63]. Genome construction was performed to a minimum sequence depth of 20 × using Flye (v 2.9.3) [64] for Nanopore reads and SPAdes (v 3.15.5) [65, 66] for Illumina reads, respectively, using default parameters and subsequently polished with racon (v 1.4.20) [67] and medaka (v 1.11.3) [68]. Read mapping for polishing was performed with minimap2 (v 2.24) [69] and BWA (v 0.7.17) [70, 71], and polishing was performed using Pilon (v 1.24) [72]. One MAG was additionally polished with Polypolish (v 0.6.0) [73]. Binning was performed using Metabat2 (v 2.15) [74]. The quality of the resulting bins was assessed using CheckM2 [75] through UseGalaxy.eu (v 1.2.4) [76]. MAG annotations [77] were conducted using Bakta (v 1.9.3) [78] for bacterial MAGs and Prokka (v 1.15.6) [79] for archaeal MAGs. Circularity assessment of the MAGs consisting of one contig was performed by appending the contig to itself using SeqKit2 [80] to recreate the seam. Nanopore and Illumina reads were mapped individually against the appended contig using minimap2 [69] and SAMtools [81]. Junction-spanning reads were defined for Nanopore as reads spanning the junction (at L = original contig length) and extending at least 1000 bp in both directions from it, and for Illumina as reads where the forward and reverse pair bridged the seam. The genome was deemed circular if the number of reads spanning the seam was ≥ 1 for both analyses. Gene prediction was performed using Prodigal (v 2.6.3) [82] and tRNA genes were identified using tRNAscan-SE [83]. Functional annotation was performed using eggNOG-mapper (v 2.1.9) [84]. Taxonomic assignments of MAGs were made using Sourmash (v 4.8.5) [85] and GTDB-tk (v 2.4.3) [86]. Additional KO numbers for prediction of translated proteins were obtained using BlastKoala [87]. For higher-resolution annotation of hydrogenases, coding sequences were classified against the curated hydrogenase database HydDB (v 1.0) [88], and true positives were confirmed by screening for conserved FeFe and NiFe binding motifs following Weng et al. [44]. Signal peptides were predicted using SignalP (v 6.0) [89] in fast mode with the organism type set to "other", while subcellular localizations were determined via DeepLocPro (v 1.0) [90] using an organism group setting of "archaea" or "any" for archaeal and bacterial genomes, respectively. The β-oxidation pathway was annotated by Bakta and cross-validated through sequence-specific analyses using KEGG [91], Uniprot [92], BLAST [61] and InterPro [93]. InterPro was deemed the most reliable database in cases where the results were not consistent across databases.

Genes associated with the β-oxidation pathway, ammonia resistance, the Wood–Ljungdahl pathway, reductive glycine pathway, glycine reductase synthase pathway and hydrogenotrophic methanogenesis were identified using predefined gene lists (Table S1). For each pathway step, the corresponding enzymatic functions were inferred by matching reference KO identifiers to KEGG annotations or via regex-based matching of KEGG gene symbols against functional genome annotations. For the β-oxidation, this screening was supplemented using a manually curated annotation of MAG1 as a reference. Loci in the remaining MAGs were assigned to specific β-oxidation steps if they possessed a matching KO identifier, an identical functional protein annotation, or an identical top BLASTP hit against the NCBI RefSeq Select protein database (restricted to Bacteria and Archaea) as the reference MAG1 coding sequence. Cross-referencing was performed by InterPro to account for false positives. Pathway profiles across the genomes were visualized as binary presence–absence heatmaps in R using pheatmap (v 1.0.13) [94].

The 16S rRNA sequences extracted from the binned MAGs were aligned with the corresponding region of the generated amplicon sequence variants obtained by 16S rRNA gene Illumina sequencing by BLAST to determine identity.

### Phylogenetic analyses

Taxonomic assignment was based on both the 16S rRNA gene sequence and whole-genome phylogeny, as the former enabled linkage of the MAG to the amplicon sequencing data, whereas the latter provided higher taxonomic resolution and robust assessment of phylogenetic placement and species novelty.

Whole-genome phylogeny analysis was conducted using available annotated NCBI Reference Genomes for described species in the Syntrophomonadaceae family (*Syntrophothermus lipocalidus* DSM 12680, GCF_000092405.1; *Syntrophomonas wolfei* subsp. *wolfei* str. Goettingen G311, GCF_000014725.1; *Syntrophomonas curvata*, GCF_052976825.1; *Syntrophomonas palmitatica* JCM 14374, GCF_001311885.1; *Syntrophomonas erecta*, GCF_052919835.1; *Candidatus* Syntrophocurvum alkaliphilum, GCF_009734445.1) supplemented with the GTDB-Tk match (GCA_012799365.1) closest to MAG1, and with *Eubacterium limosum* (GCF_000807675.2) as the outgroup. Orthogroup inference and multiple-sequence alignments were performed with OrthoFinder (v 3.1.0) [95] employing the MSA workflow with default parameters. The concatenated species-tree alignment comprised 755 orthogroups with single-copy representatives in at least 8 of the 9 proteomes.

A maximum-likelihood tree was inferred from the concatenated alignment with IQ-TREE (v 3.0.1) using LG + F + I + R7 as the substitution model, and branch support was assessed with 1000 bootstrap replicates.

The phylogenetic placement of the novel SBOB candidate was determined using the 16S rRNA gene extracted from its MAG and compared with publicly available 16S rRNA sequences of closely related taxa retrieved from RefSeq, using *E. limosum* as the outgroup [58]. Sequences were aligned using MAFFT (v 7.490) [96] with 1000 global-pair iterations, and poorly aligned positions were removed with trimAl (v 1.5.0) [97] using the automated1 option. A maximum likelihood tree was inferred with IQ-TREE (v 3.0.1) [98] using automatic model selection, and branch support was evaluated with 1000 ultrafast bootstrap and 1000 SH-aLRT replicates.

Digital DNA–DNA hybridisation (dDDH) values were calculated using the Genome-to-Genome Distance Calculator (v 3.0) [99], and average nucleotide identity (ANI) was computed with PyANI (v 0.2.9) [100], both with default parameters.

## Results and discussion

### Degradation dynamics, methane formation and cultivation conditions

**Fig. 1:**
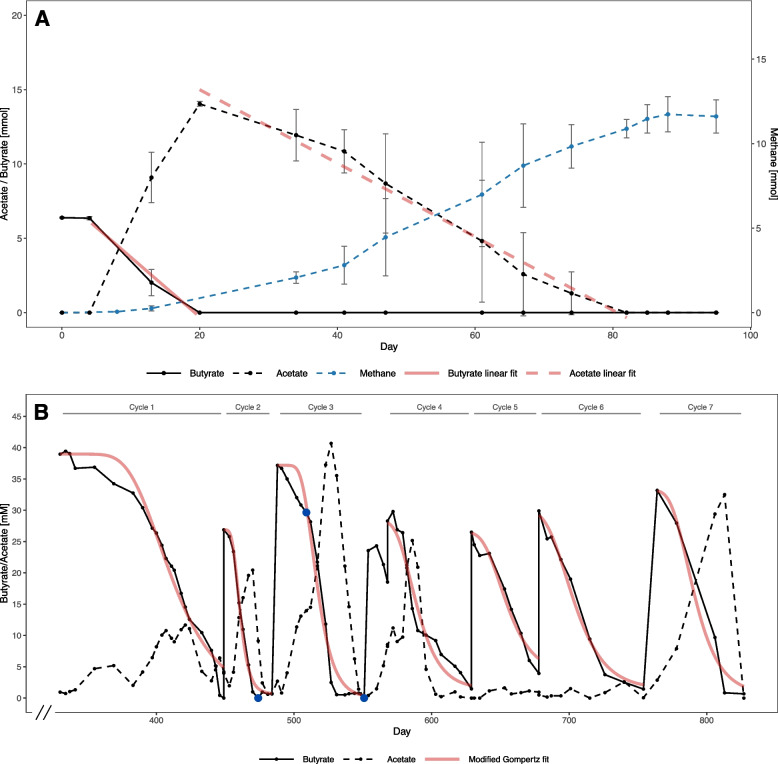
**A** Mean and standard deviation of butyrate degradation, acetate formation, acetate degradation, and methane production under thermophilic (51 ± 1 °C) conditions by cultures with yeast extract (YB). Red solid and dashed lines represent the linear fit for butyrate and acetate degradation, respectively. **B** Butyrate degradation, and acetate formation and degradation for cycles 1–7 in the thermophilic culture without yeast extract (0B). No rate calculations were performed for cycle 8 due to insufficient sampling points. Solid red lines represent the modified Gompertz model fit (Eq. 6) and blue dots represent timepoints for whole genome sequencing (day 474, 510 and 552)

The triplicate butyrate-degrading cultures supplemented with yeast extract (YB) exhibited a lag phase of approximately four days, after which butyrate was degraded at a rate of 0.40 ± 0.00 mmol/day (Fig. 1A). From the initial 6.4 ± 0.04 mmol of butyrate added, 14.0 ± 0.2 mmol acetate accumulated, while all other VFAs remained below the detection limit. Once butyrate was fully degraded, acetate was consumed at a rate of 0.25 ± 0.011 mmol/day (Fig. 1A). The sequential degradation of butyrate and acetate observed in the YB cultures resembled patterns reported for ammonia-tolerant syntrophic propionate-oxidizing cultures grown in comparable media [44, 101]. This delay may reflect either long lag phases of SAOB or a requirement for acetate to reach a threshold concentration that enables growth and enzymatic activities, potentially due to slow growth kinetics, energetic constraints, or the need to establish syntrophic partnerships. Alternatively, in the initial phase, the methanogens may not have been able to sufficiently reduce H_2_ and formate levels to those required for SAO, which, based on thermodynamic constrains, may need to be lower than those required for butyrate oxidation (Eqs. 1–2). In contrast, simultaneous degradation of butyrate and acetate directly after inoculation has been reported for SBO cultures grown without yeast extract and with a member of *Methanotrix* as the acetate consumer [102], which is known to have a high affinity for acetate [103].

The 0B culture, in which yeast extract was omitted, exhibited a prolonged initial lag phase of 252 days. Similar long lag phases have been observed in mesophilic syntrophic propionate oxidizing cultures at high ammonia [104] suggesting that one or more essential growth factors were limiting. The extended lag phase may also reflect the combined effects of high ammonia stress, the low energy yield and slow growth associated with syntrophic acid degradation, and the time required to establish effective interactions between syntrophic partners. Thereafter, the 0B culture subsequently converted butyrate to methane without substantial lag phases during the subsequent seven butyrate depletion cycles with rates of 0.12–0.47 mmol/day, with a mean of 0.24 ± 0.14 mmol/day (Fig. 1B, Table S2). In this culture, butyrate and acetate degradation occurred concurrently, as reflected by the low acetate accumulation (Fig. 1B). Hence, the acetate degradation rate could not be reliably determined.

Although acetate accumulated to a maximum of 10.3 mmol (equivalent to 40.7 mM or 2.4 g/L) in culture 0B, cycle 3 (Fig. 1B, Fig. S1), this did not appear to inhibit butyrate degradation. Similar tolerance has been reported for ammonia-stressed syntrophic propionate-oxidizing cultures, in which acetate concentrations up to 2 g/L (34 mM) had little impact on propionate degradation rates or lag phase [101]. In contrast, earlier studies on SBO cultures demonstrated acetate-dependent inhibition when comparing co- and tricultures differing in the presence of an acetoclastic methanogen [105]. However, another study found no effect of acetate scavenger on butyrate degradation rates, as comparable degradation was observed in cultures with and without the acetoclastic methanogen *Methanotrix soehngenii* [102]. Together, these observations suggest that the influence of acetate on SBO is context dependent and may vary with community composition and environmental conditions.

Stoichiometrically, during SBO, 1 mol of butyrate yields 2 mol of acetate [27] (Eq. 1). In the YB cultures, this theoretical ratio was closely reflected, with 6.4 ± 0.04 mmol butyrate yielding 14.1 ± 0.2 mmol acetate (Fig. 1A), corresponding to a butyrate-to-acetate ratio of 1:2.20. The observed excess acetate was unexpected but may have originated from the degradation of cysteine and yeast extract. Yeast extract contains amino acids, such as Ala, Gly, Asp, Glu, Ser, Thr, Cys, Tyr, Lys, and His that can be converted to acetate on a 1:1 basis [106, 107]. For the applied yeast extract (0.2 g/L) this corresponds to approximately 0.05–0.41 mmol acetate. In addition, cysteine (0.5 g/L), added as reducing agent, could theoretically yield 1 mmol acetate [108]. After correcting for these sources (1.41 mmol), the butyrate-to-acetate ratio is adjusted to 1:1.98, which is close to the theoretical stoichiometric value. Comparable studies of butyrate oxidation have reported similar values (1:1.971 [109], ~ 1:2 [102], ~ 1:1.9 [22, 105] and ~ 1:2.21 [26]). As methane formation was 0.3 ± 0.1 mmol by day 13, this indicates that some acetate was already converted prior to the observed acetate peak by day 20 (Fig. 1A).

Based on theoretical stoichiometry, the oxidation of 1 mol of butyrate is expected to yield 2.5 mol of CH_4_, comprising 2 mol derived from acetate conversion and 0.5 mol from CO_2_/H_2_ (or formate) via hydrogenotrophic methanogenesis (Eqs. 1, 2, 3, 4) [28, 110]. In the YB cultures, 6.4 ± 0.04 mmol of butyrate resulted in the production of 19.1 ± 0.7 mmol of methane [27] (Fig. 1A), corresponding to a butyrate-to-methane conversion ratio of 1:2.99. This exceeds both the theoretical value and those reported in comparable studies (1:2.33 [105], 1:2.5 [102]). However, in a study using ^13^C-labelled butyrate, ~ 80% of the butyrate-derived carbon was recovered as methane [8]. In the present study, 74.8% of the carbon from butyrate was converted to methane in the YB culture, indicating a comparable level of carbon recovery, with the remaining carbon likely incorporated into biomass. In the 0B culture, the concurrent depletion cycles resulted in a butyrate-to-methane conversion ratio of 2.09 ± 0.45 mmol CH_4_ per mmol supplemented butyrate (Table S2). During cycle 3, methane production corresponded to 1.86 mmol CH_4_ per mmol supplemented butyrate (Fig. S1). These values are closer to the theoretical stoichiometry but corresponds to a carbon conversion efficiency of only 45.9–51.6%. This lower recovery may indicate that a larger fraction of the butyrate-derived carbon was retained within the microbial community through biomass formation and other anabolic processes, as no yeast extract was supplied to provide an alternative carbon source. The headspace CO_2_ concentration ranged between 13.4–23.3% in the YB cultures and 13.1–24.9% in the 0B culture throughout all degradation cycles.

The initial pH was 7.3 in the YB medium and 7.4 in the 0B medium, providing an initial NH_3_ concentration of 0.1 g/L (Eq. 1). Upon butyrate addition, the pH of the 0B culture decreased to 7.1, but subsequently increased to 7.8–8.2 as butyrate degradation commenced and maintained, corresponding to NH_3_ concentrations of 0.3–0.5 g/L. This range coincides with the NH_3_-N transition window (0.03–0.5 g/L) previously reported to favour SAO over acetoclastic methanogenesis [4, 11].

### Community structure

**Fig. 2:**
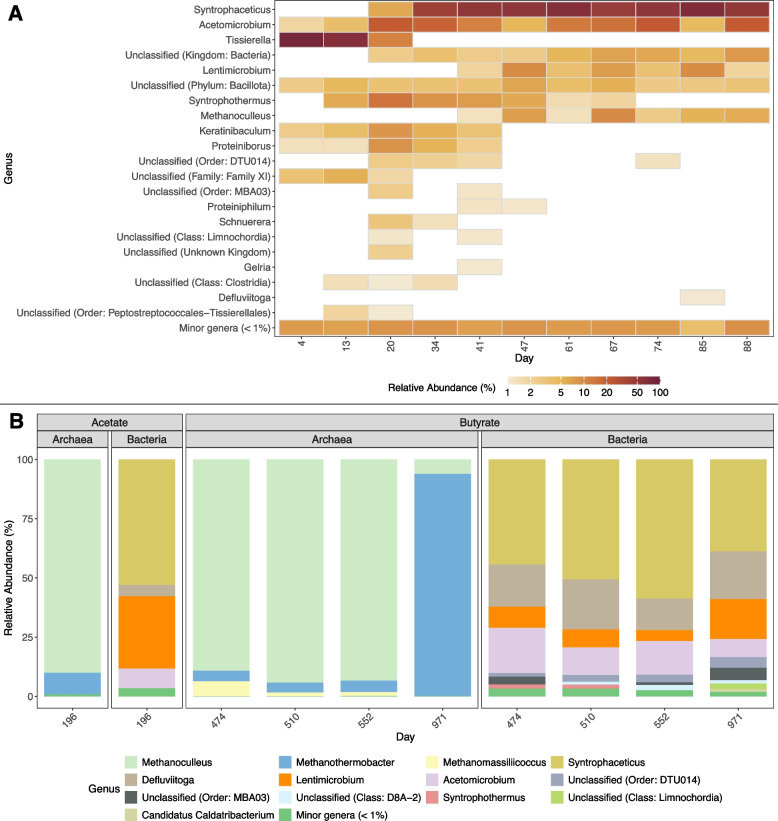
Microbial community structure based on 16S rRNA gene sequencing of cultures. **A** supplemented with yeast extract (YB, merged triplicates) and (**B**) cultures grown without yeast extract (0B), showing archaeal and bacterial communities. During visualization of panel (**B**), sequences assigned to bacteria in the archaeal study and archaea in the bacterial study were excluded

The 16S rRNA gene amplicon sequencing of the YB cultures revealed a high initial relative abundance of members of the genus *Tissierella* (Fig. 2A)*,* which declined throughout the incubation period. This trend suggests that members of this genus primarily utilized compounds derived from the yeast extract or cysteine rather than participating directly in butyrate degradation. In contrast, *Syntrophothermus* increased in relative abundance from 0 to 14% during day 4–20, corresponding to the butyrate degradation interval (Figs. 1A, 2A). *Keratinibaculum, Proteiniborus* and *Acetomicrobium* also increased during this period, each representing about 1–22% of the community (days 4–34). During the subsequent phase of acetate degradation (days 20–80), the relative abundance of *Syntrophaceticus* increased to 65% on day 61. This genus contains known mesophilic SAOB that tolerate high ammonia conditions [40]. Other genera present during acetate degradation, whose relative abundance ranged from 0.01–9% were DTU014, *Schnuerera, Proteiniphilum*, and *Lentimicrobium*, as well as some unclassified genera affiliated with Clostridia and Limnochordia at class level (Fig. 2A).

To further enrich the syntrophic butyrate-oxidizing community, yeast extract was omitted from subsequent cultivations, thereby reducing the abundance of microorganisms growing on medium-derived organic compounds. However, microorganisms utilizing compounds released through cross-feeding, cell lysis, or cysteine degradation could persist. Therefore, an acetate-fed culture lacking yeast extract was established in parallel to identify populations associated with acetate degradation and other secondary carbon sources, allowing these organisms to be distinguished from microorganisms directly involved in butyrate degradation. In both the 0B and acetate-fed cultures, high relative abundances of *Syntrophaceticus* (44–59%), *Defluviitoga* (5–21%)*, Lentimicrobium* (5–30%) and *Acetomicrobium* (8–19%) were observed (Fig. 2B). In addition, members of the uncharacterised lineages DTU014, D8A–2, and *Candidatus* Caldatribacterium represented 0.6–4% each (Fig. 2B).

A *Syntrophothermus*-affiliated species was detected in the 0B culture and was absent from the acetate-fed culture (Fig. 2B, Table S3), suggesting a role in SBO. This interpretation is further supported by the increase in its relative abundance during butyrate degradation in the YB cultures. The species accounted for approximately 2% of the community in the 0B culture on days 474 and 510, however, after 552 days of incubation of the 0B culture its relative abundance unexpectedly declined below 1%. This may be partly explained by the addition of fresh 0B medium on day 488, which could have increased the abundance of species grown on cysteine, and by the high archaeal signal at day 971, which may have masked low-abundance taxa in the 16S rRNA gene dataset (Fig. 2B). Following the addition of fresh 0B medium, members of an unclassified lineage within phylum Bacillota (class D8A–2, Fig. 2B) increased in relative abundance in the 0B culture from < 1% to 2% (Fig. 2B). By day 971, another unclassified member of phylum Bacillota (DTU014) and one belonging to the class Limnochordia (Fig. 2B) represented 4% and 5% of the bacterial community, respectively. All three taxa were also detected in the acetate-fed culture at low relative abundances < 1% (Fig. 2B, Table S3).

In the archaeal community, the amplicon sequencing revealed that members of the genus *Methanoculleus* accounted for 89–94% of the archaeal reads in both the acetate-fed culture and in the 0B culture at days 474–552 (Fig. 2B). Consistent with this, qPCR analysis showed that *Methanoculleus* maintained a relatively constant abundance of 10^5^ gene copies per ng DNA during days 474–552 (Fig. 2, Fig. S2). However, by day 971 in the 0B culture *Methanothermobacter* represented about 94% of the archaeal reads in amplicon sequencing (Fig. 2B), while *Methanoculleus* accounted for only 6%. Similarly, the qPCR revealed that *Methanothermobacter* increased from 10^3^ to 10^5^ gene copies per ng DNA between days 474–552 and day 971 in the 0B culture, while *Methanoculleus* remained at 10^5^ (Fig. 2, Fig. S2). *Methanothermobacter* has previously been suggested to be critical for efficient syntrophic propionate oxidation in the inoculum reactor [39] but in the present study the increase of this genus had no observable effect on the butyrate degradation rate (Fig. 1B, Table S2). Consistent with the methanogenic community composition, microscopic observations revealed predominantly fluorescent coccoid and rod-shaped cells (Fig. S3), characteristic of *Methanoculleus* and *Methanothermobacter*, respectively [111–113].

In the 0B culture at days 474–552, *Methanomassillicoccus* accounted for 2–6% of the archaeal community. Members of this genus are methylotrophic methanogens that produce methane by reducing methylated compounds, such as methanol, with H_2_ as electron donor [113, 114]. Their ability to consume H_2_ suggests a potential role as syntrophic partners. However, whether *Methanomassillicoccus* can maintain H_2_ partial pressures sufficiently low to support SBO and SAO under thermophilic conditions remains unclear. The presence of *Methanomassillicoccus* is particularly intriguing given emerging evidence that methyl compounds may facilitate syntrophic interactions between bacteria and methanogens. Methanol transfer has recently been proposed as an alternative interspecies metabolite exchange mechanism supporting syntrophy [115], and genes encoding alcohol dehydrogenases have been reported to be highly expressed by both methanogens and mesophilic SAOB under high-ammonia conditions [44, 104]. These observations raise the possibility that methylotrophic methanogenesis contributes to carbon and electron flow in thermophilic syntrophic communities, although the ecological significance of this pathway in the present culture requires further investigation.

### Metagenomic analyses

**Fig. 3:**
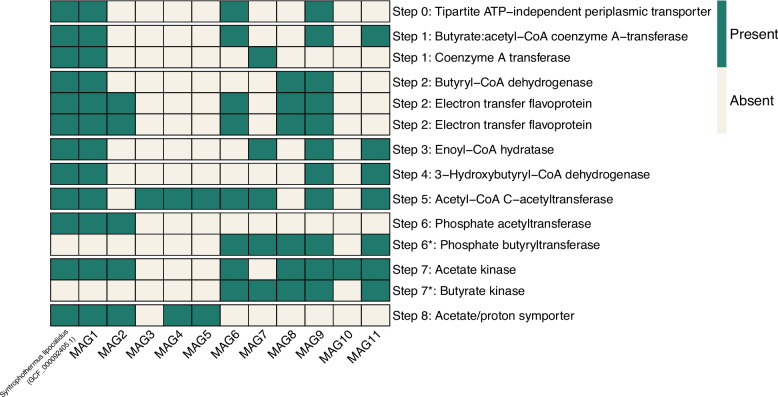
Comparison of genes involved in β-oxidation pathway between the type species *Syntrophothermus lipocalidus* (first column) and the selected MAGs (remaining columns). Rows represent individual pathway steps (Table S8–S10). Green and white cells indicate the presence and absence of genes encoding the corresponding functions, respectively. Steps marked with an * indicates a step where an alternate protein is hypothesized to hold the same function as the original one

Metagenomic binning yielded 27 annotated MAGs (Table S4–S5), and all data related to this study have been deposited in the NCBI repository PRJNA1419612. Out of these, eight MAGs were of high quality (> 90% completeness and < 5% contamination; MAG1, 3–9) [116] and three had moderate quality (> 84% completeness, < 5% contamination; MAG2, 10–11) (Table S6–S7) and were screened for presence of genes associated with the β-oxidation pathway (Fig. 3), the Wood-Ljungdahl pathway, the reductive glycine pathway and the glycine reductase synthase pathway (Fig. S4).

Of these 11 MAGs (Table S4), both MAG1 and MAG9 contained a complete set of genes associated with the β-oxidation pathway (Fig. 3). MAG1 affiliated with a lineage within the genus *Syntrophothermus* (Table S6), and its retrieved 16S rRNA gene sequence shared 100% identity with the *Syntrophothermus*-affiliated amplicon sequence detected in the 0B and YB cultures that emerged during butyrate degradation (Fig. 2). The observation that this genus was specifically affiliated with taxa enriched in butyrate-fed cultures, while remaining undetected in the acetate-fed culture (Fig. 2B), further supports the conclusion that MAG1 represents a putative SBOB in the enrichment cultures. The recovery of MAG1 further supports the presence of this species on day 552 in the 0B culture, despite its 16S rRNA gene relative abundance being below the detection limit at that time point (Fig. 2B) and its metagenomic abundance being low (Table S5). MAG9 affiliated with the phylum Bacillota, and comparison of its 16S rRNA gene sequence with the amplicon dataset linked it to the taxon classified as unclassified order DTU014 or UBA4971, depending on the reference database (Fig. 2, Table S6). This taxon was detected at low relative abundance in the acetate-fed culture (Table S3) and increased in relative abundance in both YB and 0B, particularly following acetate accumulation (DTU014 in Fig. 2). In addition to genes encoding the β-oxidation pathway, MAG9 encoded the complete reductive glycine pathway (Fig S4) and analyses demonstrated a broad metabolic repertoire. Together, these observations suggest that MAG9 may fulfil a metabolic role distinct from obligate SBO. Further investigation using approaches such as meta-transcriptomics and stable isotope probing is therefore warranted to evaluate its potential role as a SBOB. Detailed descriptions of the taxonomic placement and functional genes involved in the β-oxidation pathway of MAG1 and MAG9 are provided in subsequent sections and the supplementary information (Table S8–S12).

Other MAGs of interest for SBO interactions included MAG2, which showed high similarity to ‘*Candidatus* Thermosyntrophaceticus schinkii’ (Table S6, S13), a species previously described as a thermophilic SAOB in biogas systems [117, 118]. Owing to the relatively low completeness of MAG2, a complete Wood-Ljungdahl pathway could not be annotated (Fig. S4). However, analyses of a closely related MAG (Table S6) identified all missing genes [117], suggesting that MAG2 also possesses the genomic potential for SAO [119]. MAG7 was affiliated with an unclassified member of the family Fermentithermobacillaceae (Table S6, S14), which increased over time in 0B (D8A-2, Fig. 2) and was present at low relative abundance (< 1%) in the YB cultures. MAG8 (Table S6, S15) affiliated with the genus *Lentimicrobium* (DTU049, Fig. 2), which represented 30% and 5–17% of the bacterial community in the acetate-fed and 0B culture, respectively. MAG10 (Table S6, S16 unclassified genus DTU065) and MAG11 (Table S6, S17, unclassified order MBA03 in Fig. 2) were both related to the class Limnochordia. None of these MAGs contained a complete β-oxidation pathway (Fig. 3), but instead encoded genes associated with various amino acid and carbohydrate metabolism (Table S14–17). Furthermore, the corresponding taxa were also detected in the acetate-fed culture (Table S3), indicating that their occurrence was not exclusively linked to butyrate degradation.

**Fig. 4:**
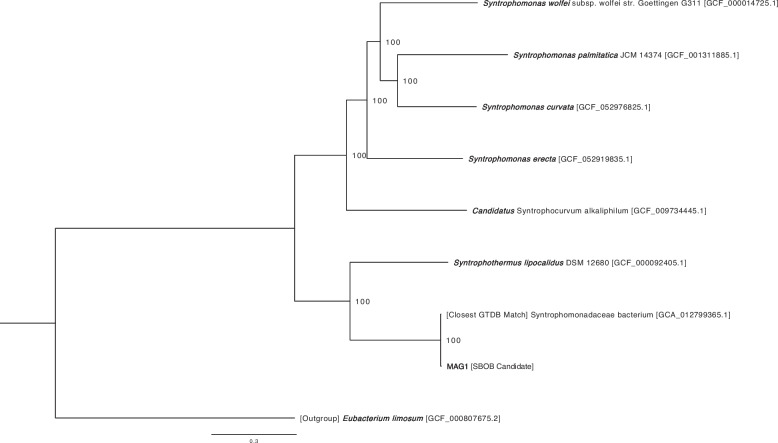
Maximum-likelihood phylogenomic tree showing the placement of MAG1 within the family Syntrophomonadaceae, based on whole-genome reference sequences from NCBI and the closest GTDB-Tk match. Numbers at nodes represent bootstrap support values (1,000 replicates)

Furthermore, MAG6, affiliated with the genus *Acetomicrobium*, is closely related to a species previously detected in both mesophilic and thermophilic syntrophic propionate- and acetate-oxidizing enrichment cultures operating at high ammonia levels (Table S6, S18–19) [39, 104]. The closest relative is *Acetomicrobium mobile*, which is known to ferment carbohydrates (e.g. glucose), organic acids (e.g. malate, pyruvate), as well as grow on yeast extract, producing acetate, H_2_, and CO_2_ [120, 121]. Based on gene expression data, this bacterium has been suggested to utilize the reductive glycine pathway and may be involved in the consumption of amino acids, formate, or acetate in syntrophic propionate-oxidizing enrichments [44, 104]. A previous study also reported a negative correlation between *Acetomicrobium* abundance and increasing butyrate concentration, indicating that it is neither favoured by nor completely inhibited by elevated butyrate levels [104, 120–122]. Another MAG that encoded most of the genes in the reductive glycine pathway was MAG11 (Fig. S4) [117, 123].

As potential H_2_- or formate-consuming partners, three methanogenic MAGs were identified: *‘Candidatus* Methanoculleus thermohydrogenotrophicum*’* (MAG3), *Methanothermobacter wolfeii* (MAG4) and *Methanothermobacter tenebrarum* (MAG5) (Table S7, S20–22). Metagenomic analyses of these MAGs revealed a complete set of genes for hydrogenotrophic methanogenesis through H_2_, CO_2_, or formate utilization (Fig. S5), consistent with the metabolic potential previously reported for these species [124, 125]. Interestingly, MAG5, representing the methanogen that increased in relative abundance at day 971 in culture 0B (Table S23), lacked genes encoding carbon monoxide dehydrogenase/acetyl-CoA synthase (CODH/ACS; Fig. S5), a key enzyme in the carbonyl branch of the Wood–Ljungdahl pathway required for CO₂ fixation into biomass by hydrogenotrophic methanogens [126]. The absence of CODH/ACS has previously been reported in species related to *M. tenebrarum* and suggested to indicate a requirement for organic carbon sources, such as acetate, for biomass synthesis [126, 127]. In culture 0B, acetate was likely continuously supplied by SBO, although this would imply competition for acetate with SAOB. No MAG of sufficient quality could be recovered for *Methanomassillicoccus* (Table S4)*.*

### The taxonomic placement of the putative SBOB (MAG1)

MAG1 had 93.73% completeness, 0.01% contamination, a genome size of 2.5 Mbp, a GC content of 49% and 2041 protein-coding sequences (Table S8). MAG1 contained six copies of the 16S rRNA gene, and phylogenetic analysis based on 16S rRNA gene sequences indicated that the closest characterized relative is *Syntrophothermus lipocalidus* (92% similarity based on BLAST searches). Accordingly, the maximum-likelihood phylogenetic tree based on 16S rRNA gene sequences identified *S. lipocalidus* as the closest characterized species (Fig. S6). *S. lipocalidus* is a thermophilic syntrophic iso-butyrate–oxidizing bacterium isolated from an up-flow anaerobic sludge blanket reactor [21].

Phylogenomic analysis of MAG1 and all available reference genomes within the family Syntrophomonadaceae identified 755 shared single-copy orthologous genes present in at least 88.9% of the analyzed genomes. Genome-based comparison against the GTDB reference database and the phylogenomic tree revealed high similarity between MAG1 and a MAG annotated as a Syntrophomonadaceae bacterium [GCA_012799365.1] (dDDH 94%, ANI 99%, Fig. 4, Fig. S7), previously recovered from a large-scale screening of anaerobic digesters [128]. However, the metabolic potential of this bacterium was not characterized in that study.

Based on the phylogenetic placement and ANI and dDDH values, the closest characterized taxa to MAG1 were *Thermosyntropha lipolytica* (ANI 32%, dDDH 69%, Fig. S7), a thermophilic and alkalitolerant SBOB, and the mesophilic SBOB *Syntrophothermus wolfei* (ANI 23%, dDDH 71%, Fig. S7). These low similarity levels indicate that MAG1 represents a novel and distinct lineage, phylogenetically separate from all previously characterized species. Accordingly, following the SeqCode guidelines [129] we propose the provisional name *‘Candidatus* Syntrophothermus ammoniitolerans’ for MAG1.

### The β-oxidation pathway and transport of butyrate and acetate

**Fig. 5:**
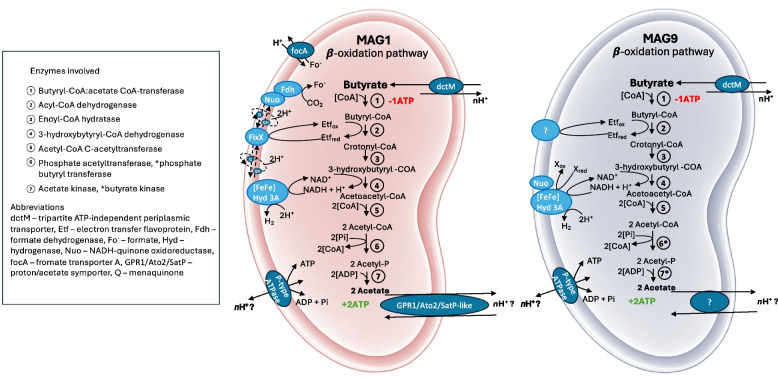
Illustration of the β-oxidation pathway inferred from the genomic potential of the putative SBOB *´Candidatus* Syntrophothermus ammoniitolerans´ (MAG1), as well as the suggested pathway of MAG9

The genetic repertoire of MAG1 further strengthened its involvement in SBO, as it encoded a complete set of genes for the β-oxidation pathway (Fig. 5, Table S10). For ATP-dependent activation of butyrate to butyryl-CoA, MAG1 encoded butyryl-CoA:acetate CoA-transferase. MAG1 also encoded Coenzyme A transferase, a general enzyme involved in CoA transfer, which may also contribute to butyrate activation. In the subsequent step, butyryl-CoA is oxidized to crotonyl-CoA by an acyl-CoA dehydrogenase. During this reaction, electrons can be transferred to electron transfer flavoprotein (EtfA/B, FixA/B family); accordingly, genes encoding this protein complex were also found in MAG1. Next, crotonyl-CoA is converted to (S)−3-hydroxybutanoyl-CoA by enoyl-CoA hydratase, followed by conversion to acetoacetyl-CoA by 3-hydroxybutyryl-CoA dehydrogenase. In step 5, an additional CoA is needed for conversion of acetoacetyl-CoA to two acetyl-CoA molecules, which in step 6 is further converted to two acetyl-P by phosphate acetyltransferase. In the final step, two ATPs and two acetate molecules are generated by substrate-level phosphorylation [39, 44, 130]. The genes encoding enzymes involved in a key catabolic pathway were organized in an operon-like cluster in MAG1. Specifically, most genes encoding the β-oxidation pathway were co-localized, except for those encoding steps 7–8 and the proposed import and export proteins, respectively (Table S8–10). Such gene clustering is considered advantageous for syntrophic microorganisms, as coordinated expression of functionally related genes reduces the energetic costs associated with transcription and facilitates efficient regulation under the low-energy conditions characteristic of syntrophic metabolism [130].

MAG9 also encoded genes for all steps of the β-oxidation pathway but, unlike MAG1, the corresponding genes were dispersed throughout the genome rather than organized in an operon-like cluster. Furthermore, no canonical phosphate acetyltransferase (Pta) was identified (Fig. 3, Table S12). Although KEGG annotated PlsX as Pta, its location within a conserved phospholipid biosynthesis gene cluster rather indicates a likely role in lipid metabolism [131]. Furthermore, MAG9 encoded putative Pta/Ptb and Buk proteins, although their predicted functions could not be unambiguously assigned based on sequence analysis.

Both MAG1 and MAG9 encoded genes for a membrane-associated tripartite ATP-independent transporter (TRAP), which was identified as the butyrate importer in the mesophilic SBOB *S. wolfei* [132]. Amino acid identity to the *S. wolfei* subunits ranged from 51–78% for MAG1, whereas for MAG9 one subunit showed no significant similarity, and the remaining subunits ranged from 27–31%. MAG1 also encoded genes annotated as an acetate transporter (Table S10), with 71% similarity to the acetate transporter in *S. wolfei*, which was not found in MAG9. This protein has been suggested to export acetate during syntrophic growth conditions, when acetate production is high. This symport system may therefore contribute to proton motive force generation across the cytoplasmic membrane, supporting reverse electron transfer processes [133].

### Energy conservation systems and reverse electron transfer

A particularly thermodynamically constrained step in β-oxidation is the conversion of butyryl-CoA to crotonyl-CoA (step 2, Fig. 5). In this reaction, electron-transferring flavoproteins (Etf) serve as electron acceptors, coupling to a menaquinone loop that generates a relatively high reduction potential (E°′ ≈ − 10 mV) [134]. To transfer these electrons further to H_2_ or formate, the SBOB must invest energy via reverse electron transport. This could be achieved by generating a proton motive force across the inner membrane either at the expense of ATP [134, 135] or by coupling the export of formate to proton translocation via a formate transporter (focA) previously described in a syntrophic propionate-oxidizing species [136]. This mechanism may also operate in MAG1, which encoded the focA gene (Table S11).

FocA has been described as a membrane protein that facilitates formate translocation across the cytoplasmic membrane [137]. In close proximity to focA, MAG1 encoded a formate dehydrogenase subunit (FdhF) and a bifurcating [FeFe]-type A3 hydrogenase, containing subunits homologs to the NADH-quinone oxidoreductase (NuoEF, Table S11). This genomic arrangement suggests the presence of energy-conserving electron transfer mechanisms similar to those described in *S. wolfei*, where Etf-mediated electron transfer is coupled with NADH produced during the conversion of 3-hydroxybutyryl-CoA to acetoacetyl-CoA by 3-hydroxybutyryl-CoA dehydrogenase (step 4, Fig. 5). Such an arrangement would provide MAG1 with flexibility to channel electrons through the most favourable carrier depending on environmental conditions and on whether its partner methanogen is able to consume formate, H_2_, or both [27].

In addition, MAG1 contained gene clusters encoding two membrane-bound formate dehydrogenases. One of these clusters (Fdh1, Table S11), included genes homologous to the cytochrome b-dependent, selenocysteine-containing formate dehydrogenase and its maturation protein FdhE (30–43% protein identity to homologs in other syntrophs, Table S11), a feature predicted to be characteristic of syntrophic bacteria [25]. Furthermore, clusters encoding [FeFe]-type C1 and C3 and genes associated with [NiFe]-type hydrogenases were identified (Table S11). Future metatranscriptomic analyses could determine whether *‘Ca.* S. ammoniitolerans’ resembles the SAOB species *S. schinkii* and *T. phaeum,* expressing both [FeFe] and [NiFe] hydrogenases in syntrophic cultures [44, 119], or the SBOB *S. fumaroxidans* and the ammonia-tolerant syntrophic propionate oxidizer candidate *Ca*. S. ammoniitolerans foremost expressing genes for [FeFe]-hydrogenases in syntrophic culture [44, 138].

In contrast to MAG1, MAG9 lacked canonical genes associated with electron transfer systems, making its energy conservation strategy less clear. Even though MAG9 encoded Etf and several [FeFe]-hydrogenases, it lacked genes encoding formate dehydrogenases. Furthermore, it lacked the FixC/FixX complex and quinones, both proposed to play key roles in reverse electron transport during syntrophic butyrate oxidation [139] and no obvious alternative mechanism for reverse electron transport was identified. Consequently, the role of MAG9 as an SBOB remains uncertain. Nevertheless, MAG9 encoded mvhD and a heterodisulfide reductase complex (HdrABC), an Rnf complex (ferredoxin:NADH reductase; rnfA–E and rnfG) located adjacent to nuoEF and a bifurcating [FeFe]-type A3 hydrogenase, suggesting the potential for energy conservation through electron bifurcation.

In regard of energy gain in SBOB, 1 ATP is most likely consumed in the first step and two ATP are generated in the last step, the net ATP yield of the β-oxidation is low (≤ 1 ATP per molecule of butyrate), depending on the mechanism of proton export and electron disposal.

### Genes associated with ammonia tolerance in the MAGs

Microorganisms employ various strategies to tolerate ammonia stress. To identify genes potentially involved in adaptation to elevated ammonia concentrations and the associated osmotic stress, metagenomes of the putative SBOB (MAG1) and known SBOB not associated with high-ammonia environments were compared (Fig. S8). Formation of multicellular aggregates that create protective microenvironments has been proposed as an ammonia tolerance mechanism [140]. While mesophilic ammonia-tolerant syntrophic cultures commonly form flocs whose disruption impairs syntrophic metabolism [44, 101, 104] no visible floc formation was observed in the thermophilic SBOB enrichment, suggesting that other tolerance mechanisms predominate.

A common initial microbial response to hyperosmotic stress is the intracellular accumulation of potassium ions (K^+^), often accompanied by counterions such as glutamate, while sodium ions (Na^+^) are exported from the cell to maintain osmotic balance [141, 142]. MAG1 encoded the high-affinity potassium uptake system Trk, however, this system was also present in other characterized SBOB and therefore does not appear to represent a unique adaptation (Fig. S8).

Accumulation of compatible solutes represents another widespread mechanism for coping with elevated osmolarity. Compatible solutes are highly water-soluble organic osmolytes that can be accumulated to high intracellular concentrations without interfering with cellular metabolism. In addition to maintaining osmotic balance, they stabilize proteins and protect other cellular macromolecules under stress conditions [143, 144]. MAG1 and the putative SAOB (MAG2) encoded transport systems for glycine betaine uptake, while MAG1 and ‘*Ca*. M. thermohydrogenotrophicum’ (MAG3) both encoded biosynthetic pathways for proline and glutamine, all of which can function as compatible solutes (Fig. S8, Table S20). Nε-acetyl-β-lysine is a compatible solute previously shown to accumulate in *Methanoculleus bourgensis* strains originating from high-ammonia biogas digesters, where they function as syntrophic partners [145]. Interestingly, ‘*Ca*. M. thermohydrogenotrophicum’ (MAG3) was the only MAG encoding both lysine 2,3 amino mutase and lysine acetyltransferase required for Nε-acetyl-β-lysine synthesis (Table S20), suggesting that this pathway may represent an adaptation of *Methanoculleus* species to high-ammonia conditions.

A notable feature distinguishing MAG1 from known SBOB genomes was the presence of a complete ectoine biosynthesis pathway (ectA, ectB, and ectC, Fig. S8). Ectoine is a widely distributed zwitterionic compatible solute in bacteria and its biosynthesis proceeds from aspartate semialdehyde [146]. The absence of ectoine biosynthesis genes in known SBOB genomes (Fig S9) and as well the other MAGs (Table S12-S22) suggests that ectoine production may represent an adaptation contributing to the ammonia tolerance of *‘Ca.* S. ammoniitolerans’.

## Conclusion

This study identified a novel putative SBOB enriched from a high-ammonia biogas process, for which the provisional name ‘*Candidatus* Syntrophothermus ammoniitolerans’ is proposed. Metagenomic analyses revealed that this MAG encoded a complete β-oxidation pathway together with key electron transfer systems supporting syntrophic energy conservation. A second MAG (MAG9) encoded a complete β-oxidation pathway but lacked genes associated with known reverse electron transfer mechanisms, making its role as an SBOB uncertain and warrant further investigation. Microbial community analyses further indicated that acetate produced during butyrate oxidation was consumed by SAOB, while members of the genera *Methanoculleus* and *Methanothermobacter* likely served as hydrogenotrophic methanogenic partners. Together, these findings expand current knowledge of ammonia-tolerant syntrophic communities and provide new insights into the metabolic capacity and ecological interactions of SBOB in nitrogen-rich anaerobic digestion systems.

## Supplementary Information

Supplementary Material 1.

Supplementary Material 2.

## Data Availability

The sequencing data and metagenome-assembled genomes (MAGs) generated in this study have been deposited in NCBI under Bioproject PRJNA1419612 available at the following URL: https://www.ncbi.nlm.nih.gov/bioproject/PRJNA1419612/, and the described MAGs have accession numbers JBWQOP000000000, JBWQOQ000000000, JBWQOR000000000, JBWQOS000000000, JBWQOT000000000, JBWQOU000000000, JBWQOV000000000, JCAJWS000000000, JCAJWT000000000, JCAJWU000000000 and JCAJWV000000000. The accession numbers are presented in Table S24.

## Abbreviations

0B medium: Sodium butyrate-supplemented medium
ANI: Average nucleotide identity
BLAST: Basic Local Alignment Search Tool
dDDH: Digital DNA–DNA hybridisation
GTDB: Genome Taxonomy Database
MAG: Metagenome assembled genome
SAO: Syntrophic acetate oxidation
SAOB: Syntrophic acetate-oxidizing bacteria
SBO: Syntrophic butyrate oxidation
SBOB: Syntrophic butyrate-oxidizing bacteria
TRAP: ATP-independent periplasmic transporter
qPCR: Quantitative PCR
VFA: Volatile fatty acid (i.e. acetate, propionate, butyrate)
YB medium: Yeast extract- and sodium butyrate-supplemented medium
