# Supplementary material for: Metagenomic and cultivation-based description of a syntrophic butyrate-oxidizing bacterium from a thermophilic and high-ammonia biogas process
Source: BMC Microbiol. 2026 Jul 30;26:680. doi: 10.1186/s12866-026-05457-y (PMC13422317; doi:10.1186/s12866-026-05457-y)
Supplement: Supplementary file 1 — Supplementary Material 1. [file 12866_2026_5457_MOESM1_ESM.pdf]

**SUPPLEMENTARY MATERIAL**

**Title:** Metagenomic and cultivation-based description of a syntrophic butyrate-oxidizing bacterium from a thermophilic and high-ammonia biogas process

**Journal:** BMC Microbiology Springer Nature

**Authors:** Malin Tiefensee[1], Nils Weng[1], Jonas A. Ohlsson[1], Maria Westerholm[1]*

**Affiliations:** [1]Department of Molecular Sciences, Swedish University of Agricultural Sciences, Uppsala, Sweden.

*Corresponding author: Maria.Westerholm@slu.se

**Table S2.** Supplemented sodium butyrate per cycle and the corresponding degradation rates in [mmol] and [mM] for the 0B culture during cycle 1–7 as well as the methane conversion per butyrate molecule added. For data points with gas ventilation without gas sampling, the methane percentage was approximated by linear regression based on adjacent sampling points.

| Cycle | Added butyrate [mmol] | Degradation rate [mmol/day] | Degradation rate [mM/day] | Methane conversion per butyrate [mmol] |
|---|---|---|---|---|
| C1 | 9.40 | 0.12 | 0.62 | 1.84 |
| C2 | 6.07 | 0.47 | 2.21 | 2.72 |
| C3 | 10.33 | 0.40 | 1.67 | 1.85 |
| C4 | 6.96 | 0.25 | 0.88 | 2.67 |
| C5 | 5.78 | 0.12 | 0.65 | 1.77 |
| C6 | 6.49 | 0.14 | 0.69 | 2.14 |
| C7 | 6.88 | 0.17 | 0.96 | 1.67 |
| **Average** | **7.42** | **0.24** | **1.10** | **2.09** |

**Table S6.** Taxonomic classification of selected bacterial MAGs and their attributes. The attributes associated with the annotation is derived from Bakta (Predicted/annotated genes, hypothetical proteins, 16S rRNA and tRNA counts) and the ones associated with MAG quality are derived from checkm2.

| **Sample** | MAG1 | MAG2 | MAG6 | MAG7 | MAG8 | MAG9 | MAG10 comb2 | MAG11 bins.37 |
|---|---|---|---|---|---|---|---|---|
| Kingdom | Bacteria | Bacteria | Bacteria | Bacteria | Bacteria | Bacteria | Bacteria | Bacteria |
| Phylum | Bacillota | Bacillota | Synergistota | Bacillota | Bacteroidota | Bacillota | Bacillota | Bacillota |
| Class | Syntrophomonadia | Syntrophomonadia | Synergistia | Fermentithermobacillia | Bacteroidia | SHA-98 | DTU065 | Limnochordia |
| Order | Syntrophomonadales | Thermacetogeniales | Synergistales | Fermentithermobacillales | Bacteroidales | UBA4971 | DTU065 | Darwinibacteriales |
| Family | Syntrophothermaceae | Thermacetogeniaceae | Acetomicrobiaceae | Fermentithermobacillaceae | DTU049 | UBA4971 | DTU065 | Darwinibacteriaceae |
| Genus | *Syntrophothermus* | ´*Candidatus* Thermosyntrophaceticus´ | *Acetomicrobium* | UBA3907 | DTU049 | UBA4971 | DTU065 | DTU010 |
| Species | ´*Candidatus* Syntrophothermus ammoniitolerans´[1] | ´*Candidatus* Thermosyntrophaceticus schinkii[2] | *Acetomicrobium* sp012518015[3] | UBA3907 sp002391545[4] | DTU049 sp001512885[5] | UBA4971 sp900019985[6] | DTU065 sp001512545[7] | DTU010 sp002391385[8] |
| Length (bp) | 2,504,019 | 2,006,769 | 2,234,114 | 1,918,663 | 2,250,134 | 2,281,142 | 2,268,680 | 2,684,271 |
| Contigs (count) | 1 | 10 | 9 | 10 | 5 | 11 | 2 | 40 |
| Topology | Circular | NA | NA | NA | NA | NA | NA | NA |
| Completeness (%) | 93.73 | 84.11 | 99.68 | 99.58 | 99.64 | 94.97 | 84.79 | 85.44 |
| Contamination (%) | 0.01 | 0.30 | 0.75 | 0.94 | 0.12 | 0.34 | 1.44 | 4.32 |
| GC content (%) | 48.8 | 45.7 | 45.0 | 45.1 | 29.4 | 49.6 | 48.3 | 58.3 |
| Predicted genes (count) | 2,345 | 2,084 | 2,246 | 1,999 | 1,842 | 2,187 | 2,065 | 3,291 |
| Annotated genes (count) | 2,041 | 1,856 | 1,928 | 1,504 | 1,668 | 1,776 | 1,413 | 2,215 |
| Hypothetical proteins (count) | 304 | 228 | 318 | 495 | 174 | 411 | 652 | 1,076 |
| Coding density (%) | 86.3 | 87.4 | 89.9 | 88.1 | 87.1 | 86.5 | 86.5 | 85.5 |
| 16S rRNA (count) | 6 | 6 | 6 | 6 | 6 | 6 | 12 | 12 |
| tRNA (count) | 46 | 48 | 57 | 47 | 44 | 49 | 49 | 55 |

[1]Candidatus status, as suggested by the present study.

[2]Candidatus status, as previously proposed (1).
[3]An *Acetomicrobium*-affiliated species annotated in this study as Acetomicrobium sp012518015 and the ANI and dDDH against a species found in a similar study, which was classified as *A. mobile* (3).
[4–8]Placeholder name according to GTDB r226.

**Table S7.** Taxonomic classification of selected archaeal MAGs and their attributes.

| **Sample** | MAG3 | MAG4 | MAG5 |
|---|---|---|---|
| Kingdom | Archaea | Archaea | Archaea |
| Phylum | Halobacteriota | Methanobacteriota | Methanobacteriota |
| Class | Methanomicrobia | Methanobacteria | Methanobacteria |
| Order | Methanomicrobiales | Methanobacteriales | Methanobacteriales |
| Family | Methanoculleaceae | Methanothermobacteraceae | Methanothermobacteraceae |
| Genus | *Methanoculleus* | *Methanothermobacter* | *Methanothermobacter_A* |
| Species | *´Candidatus* Methanoculleus thermohydrogenotrophicum´[1] | *Methanothermobacter wolfeii* | *Methanothermobacter_A tenebrarum* |
| Length (bp) | 2,457,159 | 1,716,880 | 1,500,969 |
| Contigs (count) | 2 | 1 | 1 |
| Topology | NA | Circular | Circular |
| Completeness (%) | 91.39 | 99.95 | 99.30 |
| Contamination (%) | 3.51 | 0.13 | 0.07 |
| GC content (%) | 58.8 | 48.6 | 42.1 |
| Predicted genes (count) | 2,504 | 1,846 | 1,656 |
| Annotated genes (count) | 1,506 | 1,412 | 1,293 |
| Hypothetical proteins (count) | 998 | 443 | 363 |
| Coding density (%) | 82.9 | 90.4 | 89.3 |
| 16S rRNA (count) | 3 | 4 | 3 |
| tRNA (count) | 43 | 36 | 39 |

[1]Candidatus status, as previously proposed (2).

**Table S19.** ANI and dDDH of the assembled MAGs in comparison to the closest associated species in GTDB. The genome accession number correlates to the one used to compare with the binned MAG from the present study.

| | MAG1 | MAG2 | MAG3 | MAG4 | MAG5 | MAG6 |
|---|---|---|---|---|---|---|
| Annotated species | ´*Candidatus* Syntrophothermus ammoniitolerans´[1] | ´*Candidatus* Thermosyntrophaceticus schinkii´[2] | ´*Candidatus* Methanoculleus thermohydrogenotrophicum´[3] | *Methanothermobacter wolfei* | *Methanothermobacter_A tenebrarum* | *Acetomicrbium sp012518015/ Acetomicrobium mobile*[4] |
| Genbank accession number for reference species | GCA_012799365.1 | GCA_048546675.1 | GCA_001512375.1 | GCA_025397995.1 | GCA_023167465.1 | GCA_036668215.1 |
| ANI [%] | 99.0 | 99.0 | 99.4 | 99.3 | 99.5 | 97.3 |
| dDDH [%] | 94.0 | 94.0 | 97.6 | 96 | 98.3 | 76.6 |

[1]Candidatus status, as suggested by the present study.

[2]Candidatus status, as previously proposed (1).

[3]Candidatus status, as previously proposed (2).

[4]An *Acetomicrobium*-affiliated species annotated in this study as Acetomicrobium sp012518015 and the ANI and dDDH against a species found in a similar study, which was classified as *A. mobile* (3).

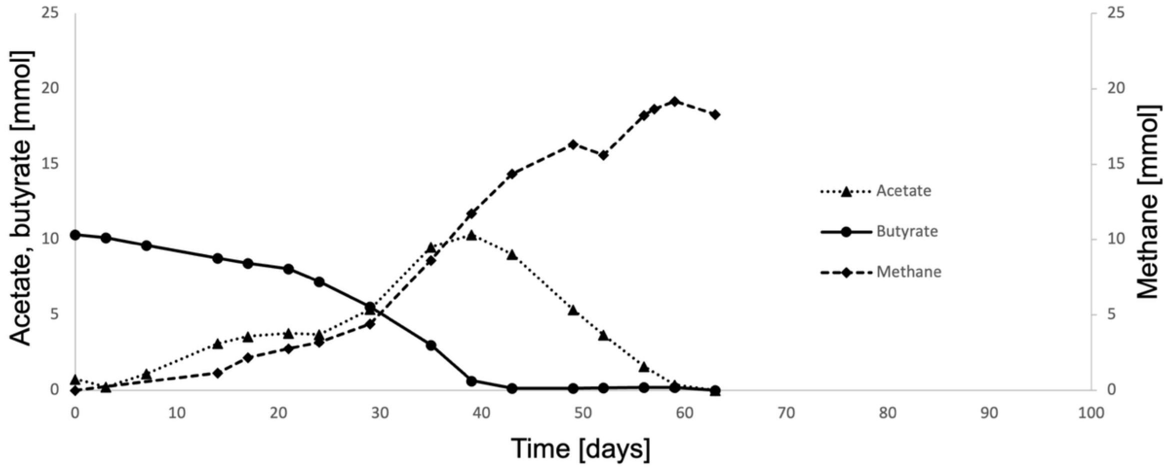


**Fig. S1:** Cycle 3 in the 0B culture. Acetate and butyrate degradation and methane accumulation over time.

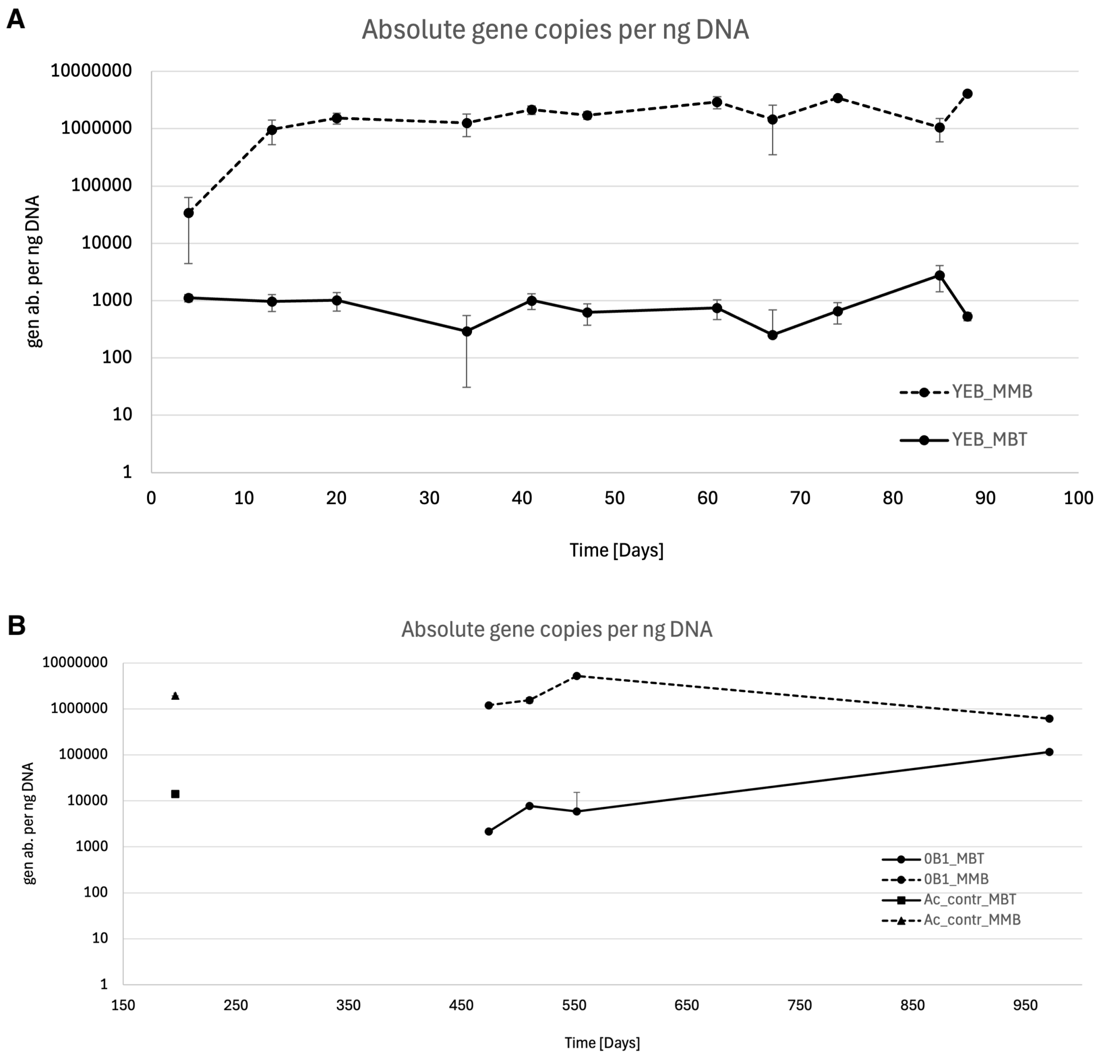


**Fig. S2.** Mean and standard deviation values of the absolute gene copies of **A** the 0B culture at day 474–971 and the acetate control culture at day 196 and the YB1-3 cultures over the course of degradation of butyrate (day 0-20) and acetate (day 20-85) (B) comparing primers targeting either *Methanoculleus* (MMB) or *Methanothermobacter* (MBT).

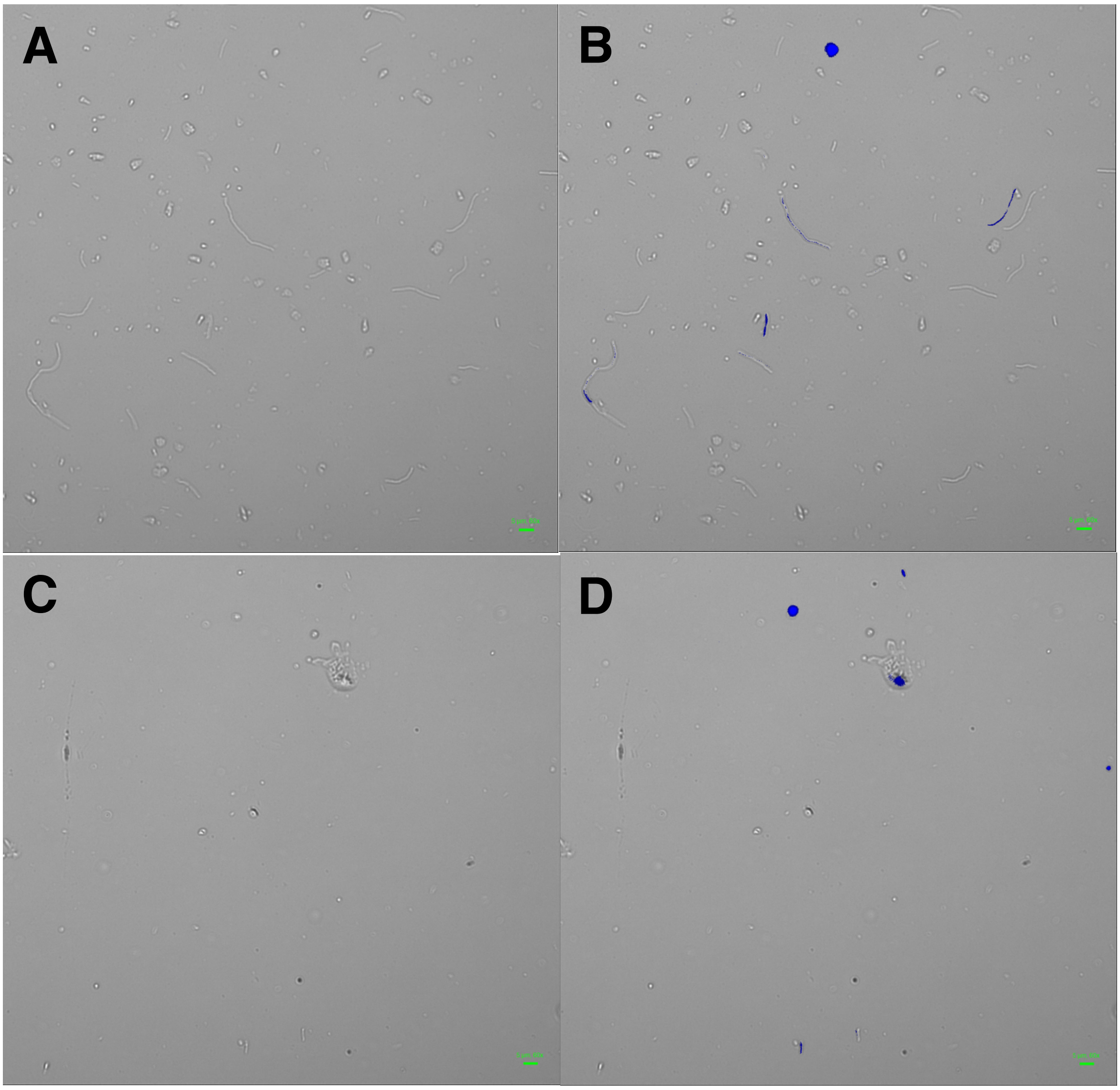


**Fig. S3.** Microscopy images of the inoculum from the propionate-fed bioreactor (A-B) and one of the YB culture triplicates (C-D). Image A, C visualize brightfield images and B, D the flourescence-brightfield composite images visualizing the shape of the methanogenic cells (blue) and the bacterial cells (uncolored). The scale bar corresponds to 5 µm, 60x.

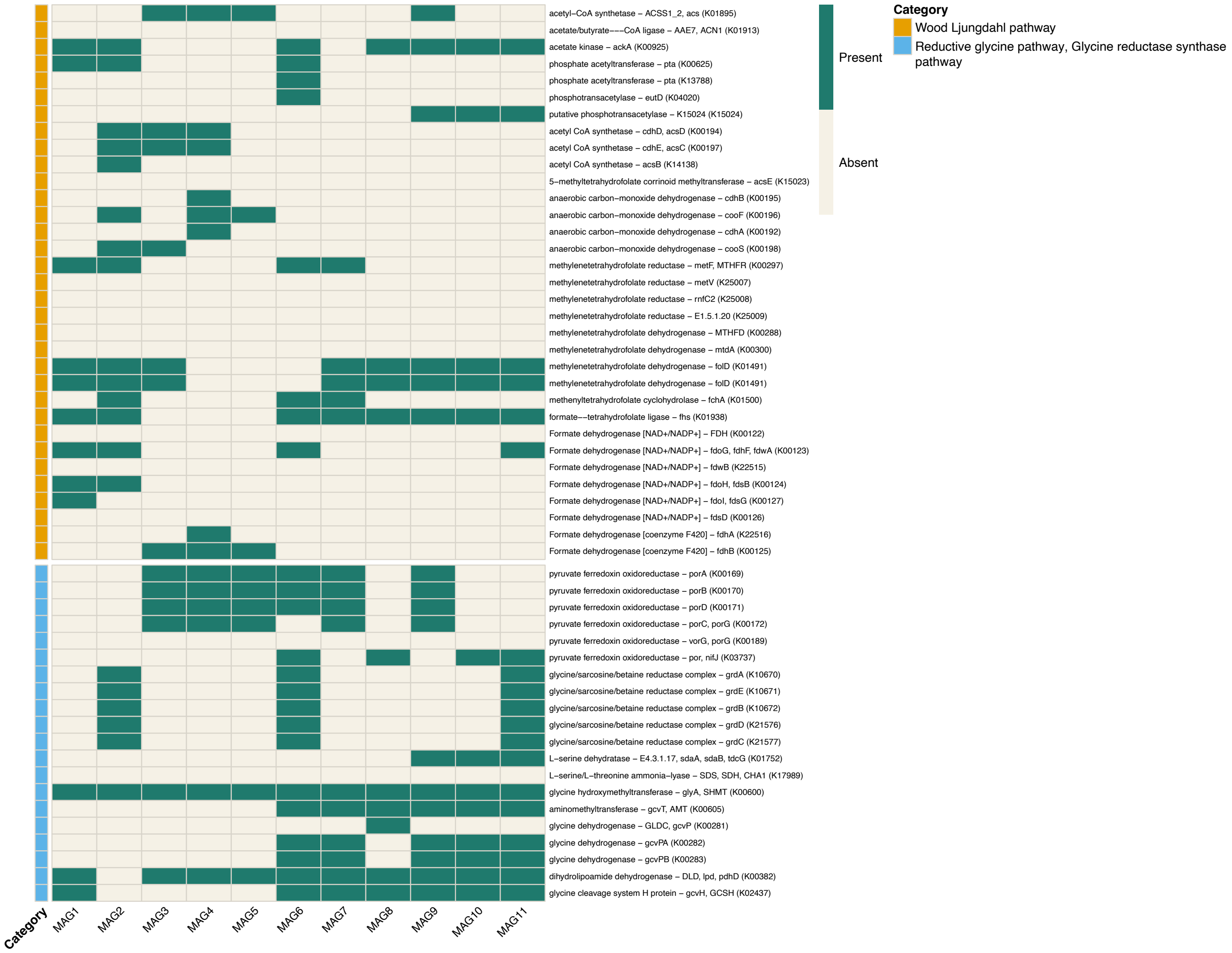


**Fig. S4.** Genes related to the Wood-Ljundahl pathway (upper), reductive glycine pathway and glycine reductase synthase pathway (lower) found in MAG1–11.

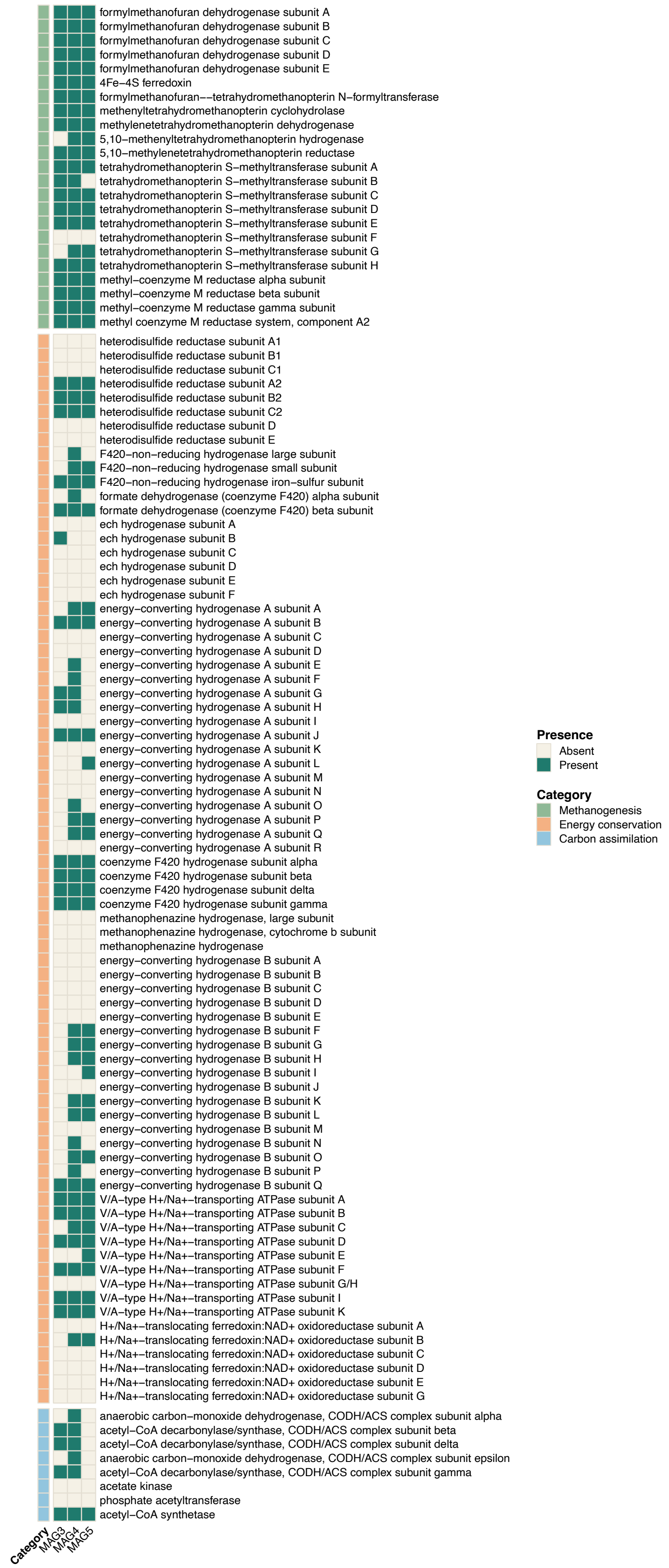


**Fig. S5.** Heatmap of methanogenesis-affiliated genes presented in Table S1 and their presence or absence in MAG3–5.

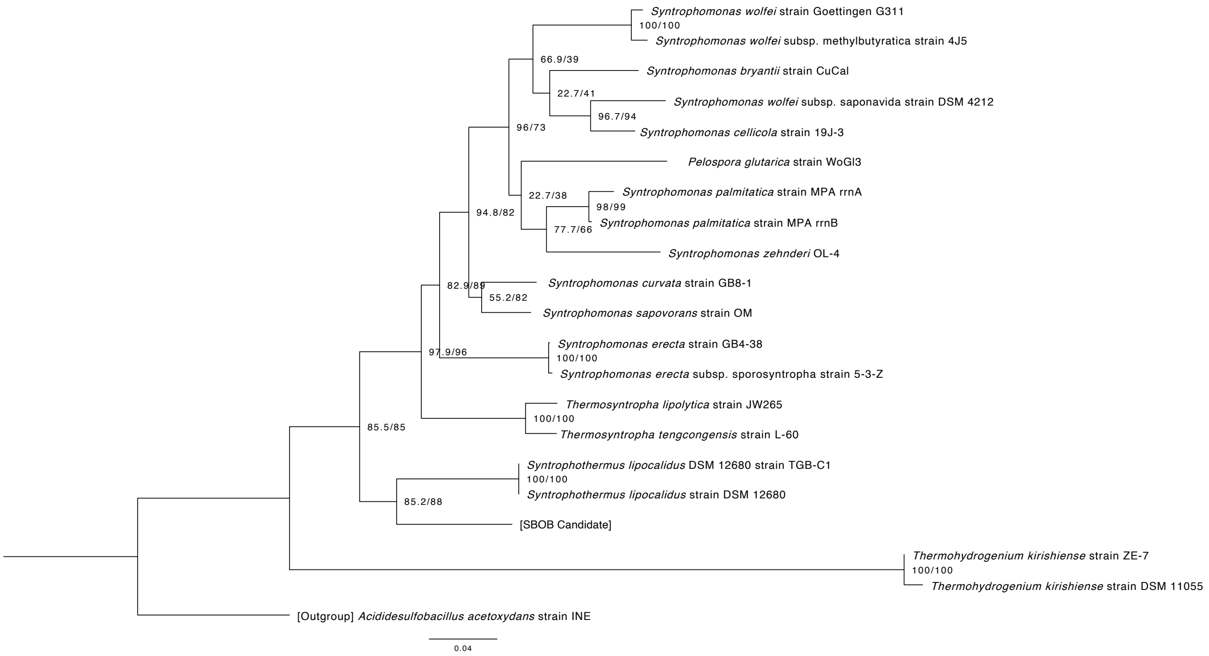


**Fig. S6.** Maximum-likelihood phylogenetic tree based on 16S rRNA gene sequences, showing the placement of the SBOB candidate (MAG1) among closely related reference taxa within the family Syntrophothermaceae. Numbers at nodes indicate ultrafast bootstrap/SH-aLRT support values.

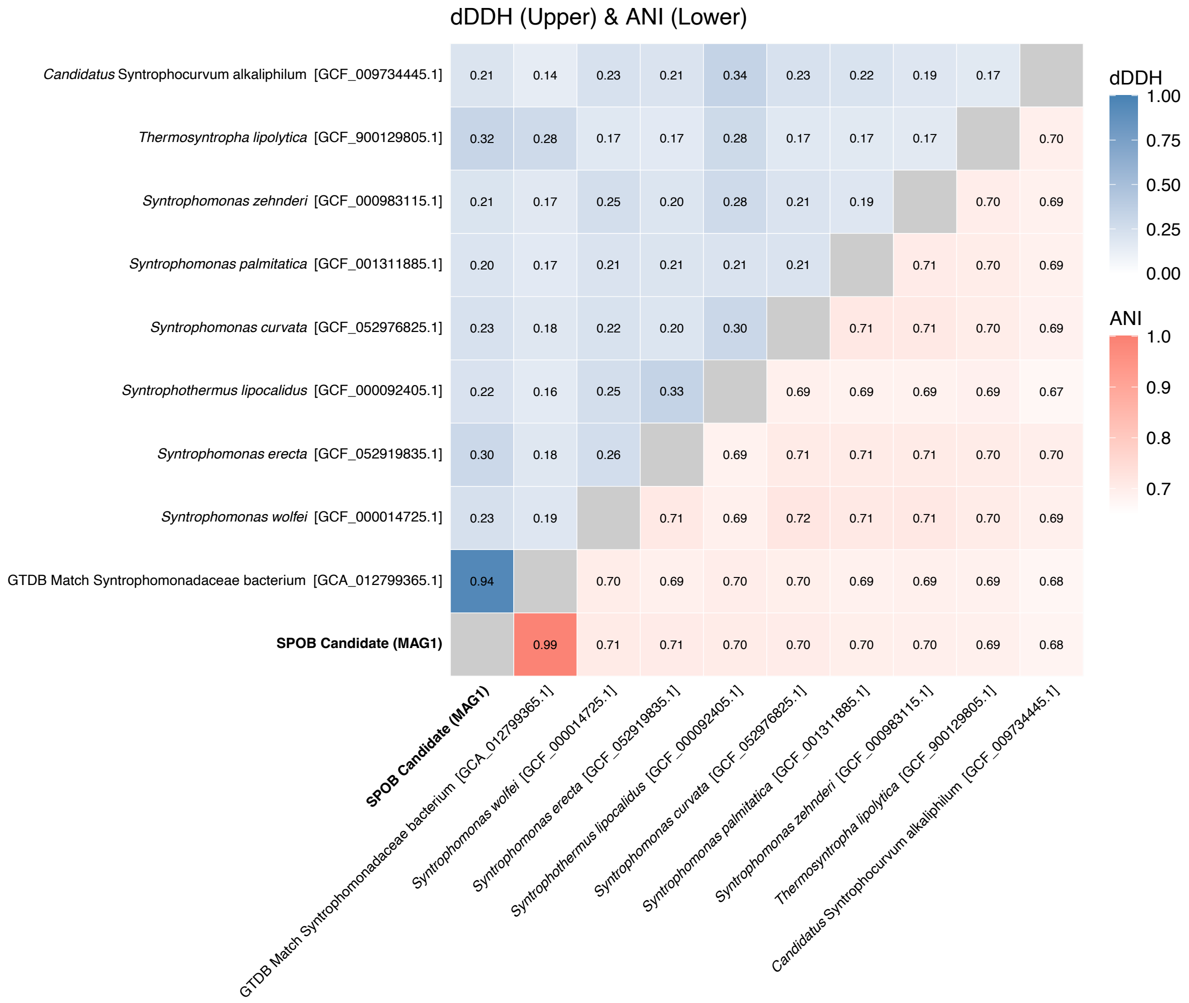


**Fig. S7.** Heatmap of dDDH and ANI percentages for the SBOB candidate (MAG1) and corresponding reference sequences.

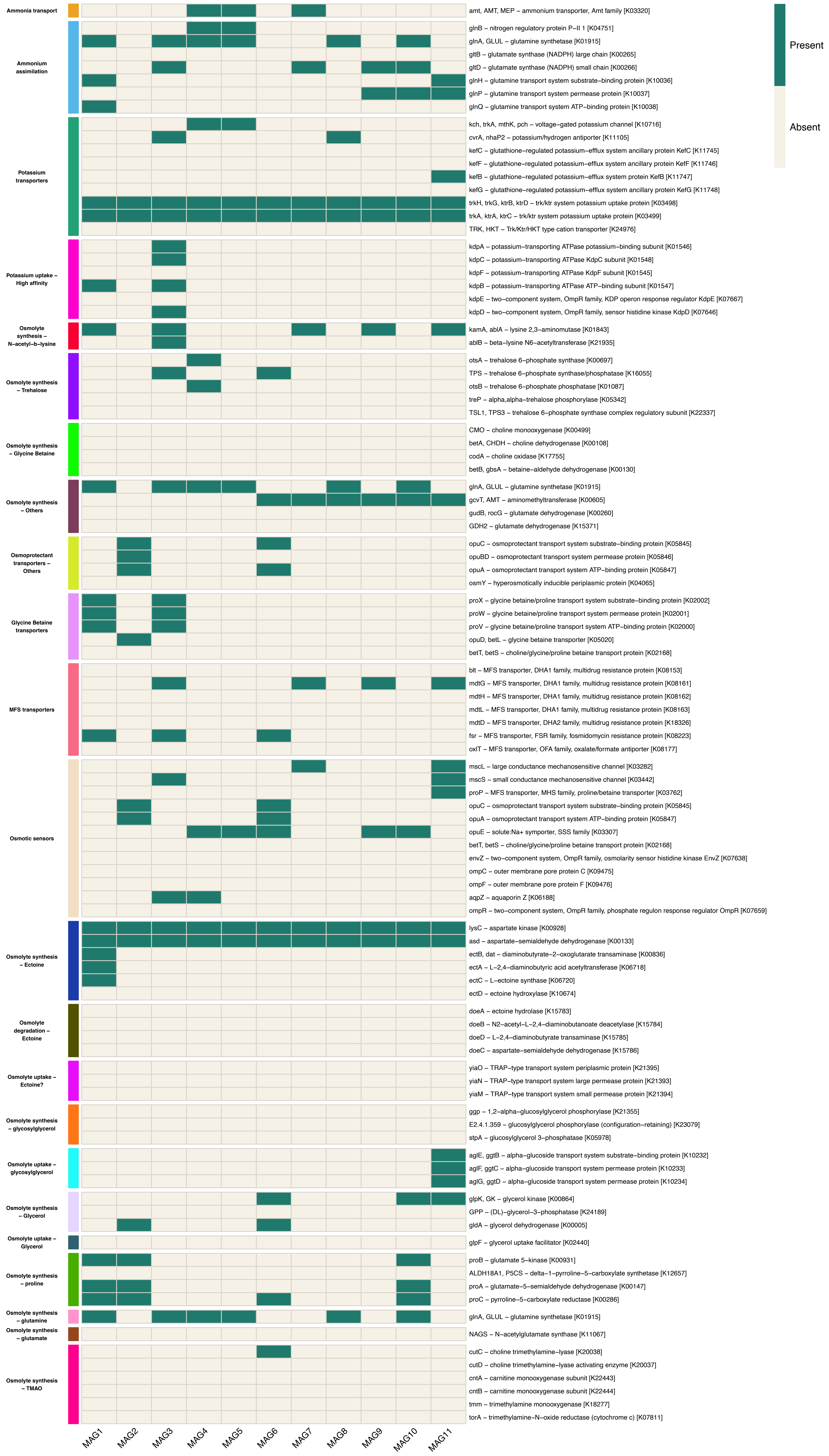
Ammonia transport
amt, AMT, MEP – ammonium transporter, Amt family [K03320]
Ammonium assimilation
glnB – nitrogen regulatory protein P–II 1 [K04751]
glnA, GLUL – glutamine synthetase [K01915]
gltB – glutamate synthase (NADPH) large chain [K00265]
gltD – glutamate synthase (NADPH) small chain [K00266]
glnH – glutamine transport system substrate–binding protein [K10036]
glnP – glutamine transport system permease protein [K10037]
glnQ – glutamine transport system ATP–binding protein [K10038]
Potassium transporters
kch, trkA, mthK, pch – voltage–gated potassium channel [K10716]
cvrA, nhaP2 – potassium/hydrogen antiporter [K11105]
kefC – glutathione–regulated potassium–efflux system ancillary protein KefC [K11745]
kefF – glutathione–regulated potassium–efflux system ancillary protein KefF [K11746]
kefB – glutathione–regulated potassium–efflux system protein KefB [K11747]
kefG – glutathione–regulated potassium–efflux system ancillary protein KefG [K11748]
trkH, trkG, ktrB, ktrD – trk/ktr system potassium uptake protein [K03498]
trkA, ktrA, ktrC – trk/ktr system potassium uptake protein [K03499]
TRK, HKT – Trk/Ktr/HKT type cation transporter [K24976]
Potassium uptake – High affinity
kdpA – potassium–transporting ATPase potassium–binding subunit [K01546]
kdpC – potassium–transporting ATPase KdpC subunit [K01548]
kdpF – potassium–transporting ATPase KdpF subunit [K01545]
kdpB – potassium–transporting ATPase ATP–binding subunit [K01547]
kdpE – two–component system, OmpR family, KDP operon response regulator KdpE [K07667]
kdpD – two–component system, OmpR family, sensor histidine kinase KdpD [K07646]
Osmolyte synthesis – N–acetyl–b–lysine
kamA, ablA – lysine 2,3–aminomutase [K01843]
ablB – beta–lysine N6–acetyltransferase [K21935]
Osmolyte synthesis – Trehalose
otsA – trehalose 6–phosphate synthase [K00697]
TPS – trehalose 6–phosphate synthase/phosphatase [K16055]
otsB – trehalose 6–phosphate phosphatase [K01087]
treP – alpha,alpha–trehalose phosphorylase [K05342]
TSL1, TPS3 – trehalose 6–phosphate synthase complex regulatory subunit [K22337]
Osmolyte synthesis – Glycine Betaine
CMO – choline monooxygenase [K00499]
betA, CHDH – choline dehydrogenase [K00108]
codA – choline oxidase [K17755]
betB, gbsA – betaine–aldehyde dehydrogenase [K00130]
Osmolyte synthesis – Others
glnA, GLUL – glutamine synthetase [K01915]
gcvT, AMT – aminomethyltransferase [K00605]
gudB, rocG – glutamate dehydrogenase [K00260]
GDH2 – glutamate dehydrogenase [K15371]
Osmoprotectant transporters – Others
opuC – osmoprotectant transport system substrate–binding protein [K05845]
opuBD – osmoprotectant transport system permease protein [K05846]
opuA – osmoprotectant transport system ATP–binding protein [K05847]
osmY – hyperosmotically inducible periplasmic protein [K04065]
Glycine Betaine transporters
proX – glycine betaine/proline transport system substrate–binding protein [K02002]
proW – glycine betaine/proline transport system permease protein [K02001]
proV – glycine betaine/proline transport system ATP–binding protein [K02000]
opuD, betL – glycine betaine transporter [K05020]
betT, betS – choline/glycine/proline betaine transport protein [K02168]
MFS transporters
blt – MFS transporter, DHA1 family, multidrug resistance protein [K08153]
mdtG – MFS transporter, DHA1 family, multidrug resistance protein [K08161]
mdtH – MFS transporter, DHA1 family, multidrug resistance protein [K08162]
mdtL – MFS transporter, DHA1 family, multidrug resistance protein [K08163]
mdtD – MFS transporter, DHA2 family, multidrug resistance protein [K18326]
fsr – MFS transporter, FSR family, fosmidomycin resistance protein [K08223]
oxlT – MFS transporter, OFA family, oxalate/formate antiporter [K08177]
Osmotic sensors
mscL – large conductance mechanosensitive channel [K03282]
mscS – small conductance mechanosensitive channel [K03442]
proP – MFS transporter, MHS family, proline/betaine transporter [K03762]
opuC – osmoprotectant transport system substrate–binding protein [K05845]
opuA – osmoprotectant transport system ATP–binding protein [K05847]
opuE – solute:Na+ symporter, SSS family [K03307]
betT, betS – choline/glycine/proline betaine transport protein [K02168]
envZ – two–component system, OmpR family, osmolarity sensor histidine kinase EnvZ [K07638]
ompC – outer membrane pore protein C [K09475]
ompF – outer membrane pore protein F [K09476]
aqpZ – aquaporin Z [K06188]
ompR – two–component system, OmpR family, phosphate regulon response regulator OmpR [K07659]
Osmolyte synthesis – Ectoine
lysC – aspartate kinase [K00928]
asd – aspartate–semialdehyde dehydrogenase [K00133]
ectB, dat – diaminobutyrate–2–oxoglutarate transaminase [K00836]
ectA – L–2,4–diaminobutyric acid acetyltransferase [K06718]
ectC – L–ectoine synthase [K06720]
ectD – ectoine hydroxylase [K10674]
Osmolyte degradation – Ectoine
doeA – ectoine hydrolase [K15783]
doeB – N2–acetyl–L–2,4–diaminobutanoate deacetylase [K15784]
doeD – L–2,4–diaminobutyrate transaminase [K15785]
doeC – aspartate–semialdehyde dehydrogenase [K15786]
Osmolyte uptake – Ectoine?
yiaO – TRAP–type transport system periplasmic protein [K21395]
yiaN – TRAP–type transport system large permease protein [K21393]
yiaM – TRAP–type transport system small permease protein [K21394]
Osmolyte synthesis – glycosylglycerol
ggp – 1,2–alpha–glucosylglycerol phosphorylase [K21355]
E2.4.1.359 – glucosylglycerol phosphorylase (configuration–retaining) [K23079]
stpA – glucosylglycerol 3–phosphatase [K05978]
Osmolyte uptake – glycosylglycerol
aglE, ggtB – alpha–glucoside transport system substrate–binding protein [K10232]
aglF, ggtC – alpha–glucoside transport system permease protein [K10233]
aglG, ggtD – alpha–glucoside transport system permease protein [K10234]
Osmolyte synthesis – Glycerol
glpK, GK – glycerol kinase [K00864]
GPP – (DL)–glycerol–3–phosphatase [K24189]
gldA – glycerol dehydrogenase [K00005]
Osmolyte uptake – Glycerol
glpF – glycerol uptake facilitator [K02440]
Osmolyte synthesis – proline
proB – glutamate 5–kinase [K00931]
ALDH18A1, P5CS – delta–1–pyrroline–5–carboxylate synthetase [K12657]
proA – glutamate–5–semialdehyde dehydrogenase [K00147]
proC – pyrroline–5–carboxylate reductase [K00286]
Osmolyte synthesis – glutamine
glnA, GLUL – glutamine synthetase [K01915]
Osmolyte synthesis – glutamate
NAGS – N–acetylglutamate synthase [K11067]
Osmolyte synthesis – TMAO
cutC – choline trimethylamine–lyase [K20038]
cutD – choline trimethylamine–lyase activating enzyme [K20037]
cntA – carnitine monooxygenase subunit [K22443]
cntB – carnitine monooxygenase subunit [K22444]
tmm – trimethylamine monooxygenase [K18277]
torA – trimethylamine–N–oxide reductase (cytochrome c) [K07811]
MAG1
MAG2
MAG3
MAG4
MAG5
MAG6
MAG7
MAG8
MAG9
MAG10
MAG11
Present
Absent

**Fig S8:** Genes related to ammonia tolerance found in MAG1–11**.**

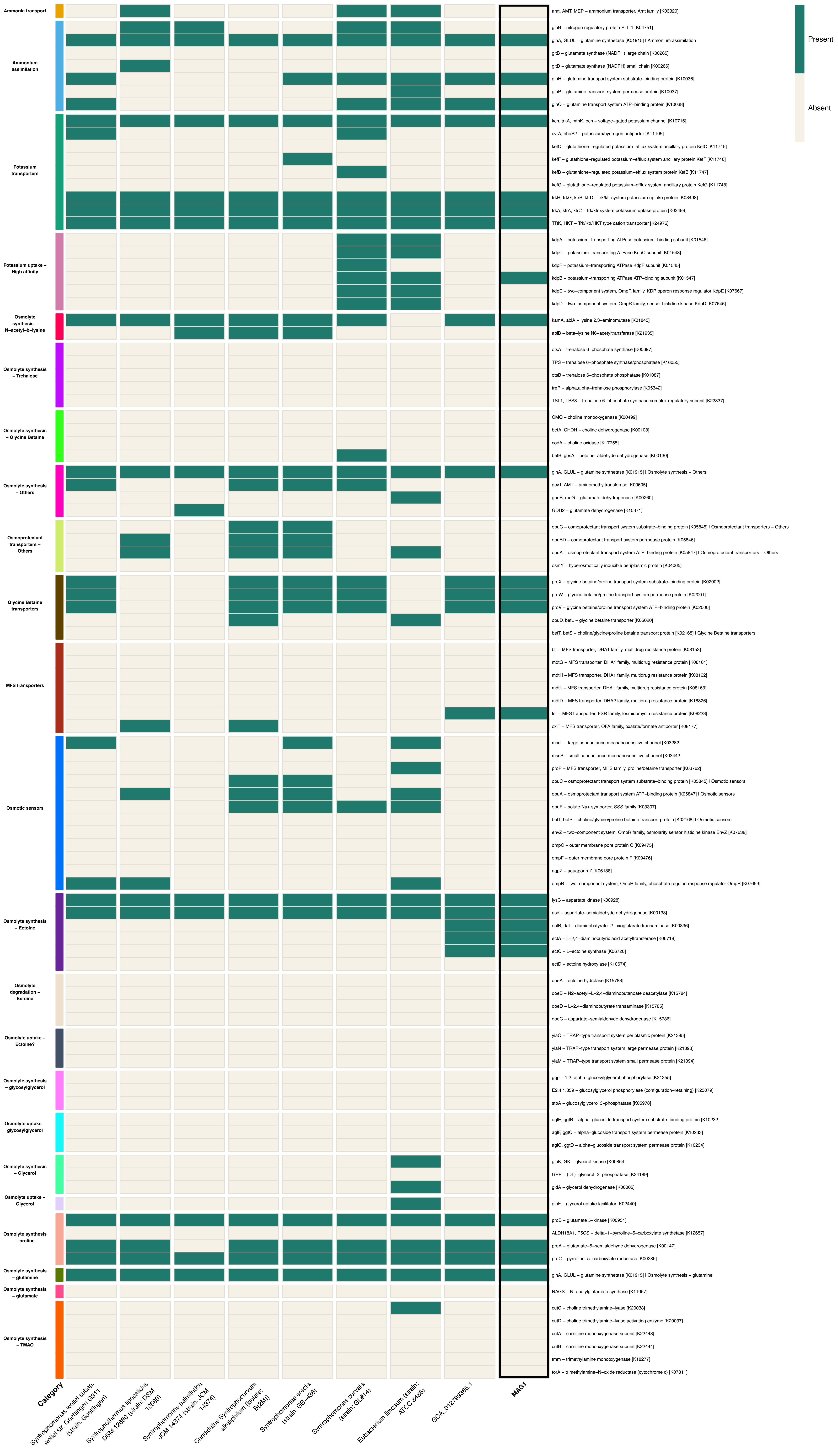

Present
Absent
Ammonia transport
Ammonium assimilation
Potassium transporters
Potassium uptake – High affinity
Osmolyte synthesis N–acetyl–b–lysine
Osmolyte synthesis – Trehalose
Osmolyte synthesis – Glycine Betaine
Osmolyte synthesis – Others
Osmoprotectant transporters – Others
Glycine Betaine transporters
MFS transporters
Osmotic sensors
Osmolyte synthesis – Ectoine
Osmolyte degradation – Ectoine
Osmolyte uptake – Ectoine?
Osmolyte synthesis – glycosylglycerol
Osmolyte uptake – glycosylglycerol
Osmolyte synthesis – Glycerol
Osmolyte uptake – Glycerol
Osmolyte synthesis – proline
Osmolyte synthesis – glutamine
Osmolyte synthesis – glutamate
Osmolyte synthesis – TMAO
amt, AMT, MEP – ammonium transporter, Amt family [K03320]
glnB – nitrogen regulatory protein P–II 1 [K04751]
glnA, GLUL – glutamine synthetase [K01915] | Ammonium assimilation
gltB – glutamate synthase (NADPH) large chain [K00265]
gltD – glutamate synthase (NADPH) small chain [K00266]
glnH – glutamine transport system substrate–binding protein [K10036]
glnP – glutamine transport system permease protein [K10037]
glnQ – glutamine transport system ATP–binding protein [K10038]
kch, trkA, mthK, pch – voltage–gated potassium channel [K10716]
cvrA, nhaP2 – potassium/hydrogen antiporter [K11105]
kefC – glutathione–regulated potassium–efflux system ancillary protein KefC [K11745]
kefF – glutathione–regulated potassium–efflux system ancillary protein KefF [K11746]
kefB – glutathione–regulated potassium–efflux system protein KefB [K11747]
kefG – glutathione–regulated potassium–efflux system ancillary protein KefG [K11748]
trkH, trkG, ktrB, ktrD – trk/ktr system potassium uptake protein [K03498]
trkA, ktrA, ktrC – trk/ktr system potassium uptake protein [K03499]
TRK, HKT – Trk/Ktr/HKT type cation transporter [K24976]
kdpA – potassium–transporting ATPase potassium–binding subunit [K01546]
kdpC – potassium–transporting ATPase KdpC subunit [K01548]
kdpF – potassium–transporting ATPase KdpF subunit [K01545]
kdpB – potassium–transporting ATPase ATP–binding subunit [K01547]
kdpE – two–component system, OmpR family, KDP operon response regulator KdpE [K07667]
kdpD – two–component system, OmpR family, sensor histidine kinase KdpD [K07646]
kamA, ablA – lysine 2,3–aminomutase [K01843]
ablB – beta–lysine N6–acetyltransferase [K21935]
otsA – trehalose 6–phosphate synthase [K00697]
TPS – trehalose 6–phosphate synthase/phosphatase [K16055]
otsB – trehalose 6–phosphate phosphatase [K01087]
treP – alpha,alpha–trehalose phosphorylase [K05342]
TSL1, TPS3 – trehalose 6–phosphate synthase complex regulatory subunit [K22337]
CMO – choline monooxygenase [K00499]
betA, CHDH – choline dehydrogenase [K00108]
codA – choline oxidase [K17755]
betB, gbsA – betaine–aldehyde dehydrogenase [K00130]
glnA, GLUL – glutamine synthetase [K01915] | Osmolyte synthesis – Others
gcvT, AMT – aminomethyltransferase [K00605]
gudB, rocG – glutamate dehydrogenase [K00260]
GDH2 – glutamate dehydrogenase [K15371]
opuC – osmoprotectant transport system substrate–binding protein [K05845] | Osmoprotectant transporters – Others
opuBD – osmoprotectant transport system permease protein [K05846]
opuA – osmoprotectant transport system ATP–binding protein [K05847] | Osmoprotectant transporters – Others
osmY – hyperosmotically inducible periplasmic protein [K04065]
proX – glycine betaine/proline transport system substrate–binding protein [K02002]
proW – glycine betaine/proline transport system permease protein [K02001]
proV – glycine betaine/proline transport system ATP–binding protein [K02000]
opuD, betL – glycine betaine transporter [K05020]
betT, betS – choline/glycine/proline betaine transport protein [K02168] | Glycine Betaine transporters
blt – MFS transporter, DHA1 family, multidrug resistance protein [K08153]
mdtG – MFS transporter, DHA1 family, multidrug resistance protein [K08161]
mdtH – MFS transporter, DHA1 family, multidrug resistance protein [K08162]
mdtL – MFS transporter, DHA1 family, multidrug resistance protein [K08163]
mdtD – MFS transporter, DHA2 family, multidrug resistance protein [K18326]
fsr – MFS transporter, FSR family, fosmidomycin resistance protein [K08223]
oxlT – MFS transporter, OFA family, oxalate/formate antiporter [K08177]
mscL – large conductance mechanosensitive channel [K03282]
mscS – small conductance mechanosensitive channel [K03442]
proP – MFS transporter, MHS family, proline/betaine transporter [K03762]
opuC – osmoprotectant transport system substrate–binding protein [K05845] | Osmotic sensors
opuA – osmoprotectant transport system ATP–binding protein [K05847] | Osmotic sensors
opuE – solute:Na+ symporter, SSS family [K03307]
betT, betS – choline/glycine/proline betaine transport protein [K02168] | Osmotic sensors
envZ – two–component system, OmpR family, osmolarity sensor histidine kinase EnvZ [K07638]
ompC – outer membrane pore protein C [K09475]
ompF – outer membrane pore protein F [K09476]
aqpZ – aquaporin Z [K06188]
ompR – two–component system, OmpR family, phosphate regulon response regulator OmpR [K07659]
lysC – aspartate kinase [K00928]
asd – aspartate–semialdehyde dehydrogenase [K00133]
ectB, dat – diaminobutyrate–2–oxoglutarate transaminase [K00836]
ectA – L–2,4–diaminobutyric acid acetyltransferase [K06718]
ectC – L–ectoine synthase [K06720]
ectD – ectoine hydroxylase [K10674]
doeA – ectoine hydrolase [K15783]
doeB – N2–acetyl–L–2,4–diaminobutanoate deacetylase [K15784]
doeD – L–2,4–diaminobutyrate transaminase [K15785]
doeC – aspartate–semialdehyde dehydrogenase [K15786]
yiaO – TRAP–type transport system periplasmic protein [K21395]
yiaN – TRAP–type transport system large permease protein [K21393]
yiaM – TRAP–type transport system small permease protein [K21394]
ggp – 1,2–alpha–glucosylglycerol phosphorylase [K21355]
E2.4.1.359 – glucosylglycerol phosphorylase (configuration–retaining) [K23079]
stpA – glucosylglycerol 3–phosphatase [K05978]
aglE, ggtB – alpha–glucoside transport system substrate–binding protein [K10232]
aglF, ggtC – alpha–glucoside transport system permease protein [K10233]
aglG, ggtD – alpha–glucoside transport system permease protein [K10234]
glpK, GK – glycerol kinase [K00864]
GPP – (DL)–glycerol–3–phosphatase [K24189]
gldA – glycerol dehydrogenase [K00005]
glpF – glycerol uptake facilitator [K02440]
proB – glutamate 5–kinase [K00931]
ALDH18A1, P5CS – delta–1–pyrroline–5–carboxylate synthetase [K12657]
proA – glutamate–5–semialdehyde dehydrogenase [K00147]
proC – pyrroline–5–carboxylate reductase [K00286]
glnA, GLUL – glutamine synthetase [K01915] | Osmolyte synthesis – glutamine
NAGS – N–acetylglutamate synthase [K11067]
cutC – choline trimethylamine–lyase [K20038]
cutD – choline trimethylamine–lyase activating enzyme [K20037]
cntA – carnitine monooxygenase subunit [K22443]
cntB – carnitine monooxygenase subunit [K22444]
tmm – trimethylamine monooxygenase [K18277]
torA – trimethylamine–N–oxide reductase (cytochrome c) [K07811]
Category
Syntrophomonas wolfei subsp. wolfei str. Goettingen G311 (strain: Goettingen)
Syntrophothermus lipocalidus DSM 12680 (strain: DSM 12680)
Syntrophomonas palmitatica JCM 14374 (strain: JCM 14374)
Candidatus Syntrophocurvum alkaliphilum (isolate: B(2M))
Syntrophomonas erecta (strain: GB–438)
Syntrophomonas curvata (strain: GL#14)
Eubacterium limosum (strain: ATCC 8486)
GCA_012799365.1
MAG1

**Fig S9:** Genes related to ammonia tolerance found in MAG1 in relation to genes present in other known SBOB originating from systems of lower ammonia levels.

## References


1. Cheng GB, Bongcam-Rudloff E, Schnürer A. Metagenomic Exploration Uncovers Several Novel 'Candidatus' Species Involved in Acetate Metabolism in High-Ammonia Thermophilic Biogas Processes. Microbial Biotechnology. 2025;18(3):e70133. doi:10.1111/1751-7915.70133

2. Kougias PG, Campanaro S, Treu L, Zhu X, Angelidaki I. A novel archaeal species belonging to *Methanoculleus* genus identified via de-novo assembly and metagenomic binning process in biogas reactors. Anaerobe. 2017 Aug 1;Biogas Science 201646:23–32. doi:10.1016/j.anaerobe.2017.02.009

3. Weng N, Singh A, Ohlsson JA, Dolfing J, Westerholm M. Catabolism and interactions of syntrophic propionate- and acetate oxidizing microorganisms under mesophilic, high-ammonia conditions. Front Microbiol. 2024 Jun 5;15. doi:10.3389/fmicb.2024.1389257
